# Genetic Screening of LCA in Belgium: Predominance of *CEP290* and Identification of Potential Modifier Alleles in *AHI1* of *CEP290*-related Phenotypes

**DOI:** 10.1002/humu.21336

**Published:** 2010-10

**Authors:** Frauke Coppieters, Ingele Casteels, Françoise Meire, Sarah De Jaegere, Sally Hooghe, Nicole van Regemorter, Hilde Van Esch, Aušra Matulevičienė, Luis Nunes, Valérie Meersschaut, Sophie Walraedt, Lieve Standaert, Paul Coucke, Heidi Hoeben, Hester Y Kroes, Johan Vande Walle, Thomy de Ravel, Bart P Leroy, Elfride De Baere

**Affiliations:** Center for Medical Genetics Ghent, Ghent University HospitalGhent, Belgium; Department of Ophthalmology, Leuven University HospitalsLeuven, Belgium; Hôpital Des Enfants Reine FabiolaBrussels, Belgium; Centre de Génétique de Bruxelles, Free University of BrusselsBrussels, Belgium; Centre for Human Genetics, Leuven University HospitalsLeuven, Belgium; Department of Human and Medical Genetics, Faculty of Medicine, Vilnius UniversityVilnius, Lithuania, Europe; Service of Medical Genetics, Hospital Dona Estefânia Rua Jacinta MartoLisboa, Portugal; Department of Radiology, Ghent University HospitalGhent, Belgium; Department of Ophthalmology, Ghent University HospitalGhent, Belgium; Revalidation Center SpermalieBruges, Belgium; Department of Nephrology, Middelheim HospitalAntwerp, Belgium; Department of Medical Genetics, University Medical Center UtrechtUtrecht, The Netherlands; Department of Pediatrics, Ghent University HospitalGhent, Belgium

**Keywords:** LCA, *CEP290*, *AHI1*, modifier, genotype-phenotype correlation

## Abstract

Leber Congenital Amaurosis (LCA), the most severe inherited retinal dystrophy, is genetically heterogeneous, with 14 genes accounting for 70% of patients. Here, 91 LCA probands underwent LCA chip analysis and subsequent sequencing of 6 genes (*CEP290, CRB1, RPE65, GUCY2D, AIPL1*and *CRX*), revealing mutations in 69% of the cohort, with major involvement of *CEP290* (30%). In addition, 11 patients with early-onset retinal dystrophy (EORD) and 13 patients with Senior-Loken syndrome (SLS), LCA-Joubert syndrome (LCA-JS) or cerebello-oculo-renal syndrome (CORS) were included. Exhaustive re-inspection of the overall phenotypes in our LCA cohort revealed novel insights mainly regarding the *CEP290*-related phenotype. The *AHI1* gene was screened as a candidate modifier gene in three patients with the same *CEP290* genotype but different neurological involvement. Interestingly, a heterozygous novel *AHI1* mutation, p.Asn811Lys, was found in the most severely affected patient. Moreover, *AHI1* screening in five other patients with *CEP290*-related disease and neurological involvement revealed a second novel missense variant, p.His758Pro, in one LCA patient with mild mental retardation and autism. These two *AHI1* mutations might thus represent neurological modifiers of *CEP290*-related disease. © 2010 Wiley-Liss, Inc.

## INTRODUCTION

Leber Congenital Amaurosis (LCA; MIM# 204000) was first described as a congenital type of retinitis pigmentosa (RP). Approximately 20% of all blind children are thought to suffer from this disease. Phenotypic features include a congenital onset, severely reduced or absent electroretinogram (ERG), nystagmus, the oculo-digital sign and a fundus aspect varying from normal to severely atrophic. Two main types of LCA have been reported, based on the presence or absence of photophobia, night blindness, hyperopia, macular/peripheral retinal abnormalities and measurable visual acuity (Hanein et al., 2004; Hanein et al., 2006). LCA displays variable expression, and seems to represent the extreme and severe end of a spectrum of inherited retinal disease.

LCA is predominantly inherited in an autosomal recessive manner. So far, one locus – *LCA9* (Keen et al., 2003) – and the following 14 genes have been identified: *GUCY2D* (Perrault et al., 1996), *RPE65* (Marlhens et al., 1997), *CRX* (Freund et al., 1998), *AIPL1* (Sohocki et al., 2000a), *RPGRIP1* (Dryja et al., 2001), *CRB1* (den Hollander et al., 2001), *RDH12* (Perrault et al., 2004), *IMPDH1* (Bowne et al., 2006), *CEP290* (den Hollander et al., 2006), *RD3* (Friedman et al., 2006), *LCA5* (den Hollander et al., 2007) and *SPATA7* (Wang et al., 2009), with the involvement of *TULP1* (Hagstrom et al., 1998) and *LRAT* (Thompson et al., 2001) under debate. Mutations in these genes account for ∼70% of all LCA cases. Several of them are also implicated in other retinal dystrophies: *CRB1, RPE65, RDH12*and *SPATA7*are associated with both LCA and early-onset retinal dystrophy (EORD), which often overlap (Gu et al., 1997; den Hollander et al., 1999; Janecke et al., 2004; Wang et al., 2009).

Several subtypes of LCA can be considered part of the ciliopathies, as four disease genes – *TULP1, RPGRIP1, CEP290* and *LCA5* – encode ciliary proteins. Since cilia are present throughout the whole body, mutations in ciliary genes may cause a broad phenotypic spectrum. One of the best examples is *CEP290*, the most frequently mutated gene in the western European LCA population. In addition to LCA, *CEP290* is associated with Joubert syndrome (JS; MIM# 213300), Senior-Loken syndrome (SLS; MIM# 266900), Meckel-Grüber syndrome (MKS; MIM# 249000) and Bardet-Biedl syndrome (BBS; MIM# 209900); a range of clinically and genetically heterogeneous ciliopathies (Sayer et al., 2006; Valente et al., 2006b; Baala et al., 2007; Brancati et al., 2007; Helou et al., 2007; Leitch et al., 2008). Recent studies suggest that modifiers may play a role in the pathogenesis of ciliopathies (Leitch et al., 2008; Khanna et al., 2009; Louie et al., 2010).

Establishing a molecular diagnosis for LCA is not only important in the context of genetic counselling and clinical prognosis, but is also essential in view of future gene therapy. Recent Phase I clinical trials for *RPE65* gene replacement therapy provide hopeful prospects for the treatment of inherited retinal dystrophies (Bainbridge et al., 2008; Hauswirth et al., 2008; Maguire et al., 2008; Cideciyan et al., 2009; Maguire et al., 2009). As such therapies are likely to be gene-specific, the development of robust clinical testing and efforts toward gene identification are of utmost importance.

Current diagnostic testing for LCA generally involves chip analysis that contains known mutations in all known LCA and EORD genes (Asper Ophthalmics, Estonia). Depending on the population, causal mutations are found in approximately 55% of all cases (Yzer et al., 2006). Only a limited number of laboratories subsequently screen an additional number of genes (Stone, 2007; den Hollander et al., 2008).

This study includes an extensive genetic survey in order to identify the molecular cause in 91 LCA probands mainly of Belgian origin, using LCA chip analysis for 8 to 13 genes and subsequent sequencing of the following genes: *CEP290* (MIM# 610142), *CRB1* (MIM# 604210), *RPE65* (MIM# 180069), *GUCY2D* (MIM# 600179), *AIPL1* (MIM# 604392) and *CRX* (MIM# 602225). In addition, exhaustive phenotyping was performed in all patients carrying mutation(s), and the *AHI1* gene was screened for modifier alleles of CEP290-related disease.

## MATERIALS AND METHODS

### Patients

Ninety-one consenting subjects initially diagnosed with LCA were referred for molecular testing by an ophthalmologist and/or geneticist, mainly associated with the University Hospitals of Ghent, Leuven or Brussels. Eleven probands are born from a consanguineous marriage. The inclusion criteria for LCA were bilateral visual loss before the age of 6 months accompanied by nystagmus and an undetectable or significantly reduced ERG. Twelve patients presented with additional mental retardation and/or autistic behaviour. For 18 patients with an available Magnetic Resonance Imaging (MRI), the absence of a molar tooth sign (MTS) excluded the diagnosis of JS. In addition, genotyping was performed on 11 probands with EORD (disease diagnosed beyond the first six months of life but before the age of three) and 13 with a retinal dystrophy in the context of JS (LCA-JS), SLS or cerebello-oculo-renal syndrome (CORS). These patients were not included during calculations of gene-specific contributions in isolated LCA. Genomic DNA and RNA were extracted from leukocytes using the Puregene DNA isolation kit (Gentra) and the RNeasy Mini kit (Qiagen) respectively, followed by cDNA synthesis with the iScript cDNA Synthesis kit (Bio-Rad). If available, parents and/or siblings were also genotyped. Seven of the patients were reported previously (Yzer et al., 2006; Brancati et al., 2007; Perrault et al., 2007). Patient notation was performed according to their clinical diagnosis (prefixes LCA, SLS, LCA-JS, CORS and EORD), with consecutive numbering in the order of the genes involved.

### Genotyping

As a pre-screening method, all patients with either isolated LCA or EORD were analysed with a microarray containing 344 to 641 mutations in 8 (*GUCY2D, CRX, RPE65, CRB1, RPGRIP1,AIPL1, LRAT* and *MERTK*) to 13 (addition of *TULP1, LCA5, RDH12, CEP290* and *SPATA7*) LCA and EORD genes (LCA chip Versions 2004–2009; Asper Ophthalmics, Estonia) (http://www.asperbio.com) (Zernant et al., 2005). Each of the mutations found by the LCA chip was subsequently confirmed through Sanger sequencing. In case of a heterozygous mutation, the coding exons and intron-exon boundaries of the involved gene were sequenced.

Patients in whom no mutations were identified after LCA chip analysis were analysed through sequencing of all coding exons and intron-exon boundaries of *CEP290, CRB1, RPE65, GUCY2D, AIPL1* and *CRX*, the first five genes being the most frequently mutated in LCA. At the time the LCA chip did not yet include *CEP290* variants, stepwise targeted mutation analysis was performed prior to sequencing of the total coding region. We initially screened for the frequent c.2991+1655A>G mutation followed by four additional mutations: c.4723A>T (p.Lys1575X), c.5587-1G>C (splice site), c.5163del (p.Thr1722GlnfsX2) and c.3310-1_3310delinsAA (splice site). The first three mutations occurred multiple times in a previous study (Perrault et al., 2007); the latter was found in three patients with a heterozygous c.2991+1655A>G mutation in our population. *CEP290* was also screened at cDNA level in patients with only a single mutation in *CEP290*. To this end, cDNA screening using 16 overlapping primer sets was optimized. Four patients with *CEP29* 0-related LCA who presented with mental retardation, two patients with SLS, one patient with CORS and one patient with LCA-JS underwent sequencing of *the AHI1* gene. For 13 patients with SLS/LCA-JS/CORS, molecular testing of *CEP290* was requested. Supp. Table S1 includes all primer sequences used in this study.

### Mutation nomenclature

Mutation nomenclature uses numbering with the A of the initiation codon ATG as +1 (http://www.hgvs.org/mutnomen), based on the following RefSeqs: NM_201253.1 (*CRB1*), NM_000329.2 (*RPE65*), NM_000180.3 (*GUCY2D*), NM_014336.3 (*AIPL1*), NM_000554.4 (*CRX*), NM_025114.3 (*CEP290*), NM_152443.2 (*RDH12)*, NM_020366.3 (*RPGRIP1)* and NM_001134831.1 (*AHI1*) (http://www.ncbi.nlm.nih.gov/nuccore). All mutations and variants found in *CEP290* were submitted to the locus-specific mutation database *CEP290base* (http://medgen.ugent.be/cep290base) (Coppieters et al., 2010).

### Evaluation of sequence changes

The presence of all mutations was confirmed on a second PCR product. Segregation analysis of disease alleles was performed if possible. Genomic DNA obtained from > 340 unrelated ethnically matched healthy individuals was used as a control panel. Thorough bio-informatic evaluation of novel variants was done using Alamut software (v.1.5). Variants were designated as “unclassified variant (UV)” if no consensus was seen in all prediction programs used. The Alamut output for missense changes is listed in Supp. Table S2.

### Clinical evaluation of patients

After identification of the molecular cause, clinical records were revisited, based on a clinical checklist comprising data on visual function, retinal appearance and associated (extra-) ocular features. When possible, ERG, fundus pictures, autofluorescence (AF) images and optical coherence tomography (OCT) were obtained. In case of CEP290-related LCA, neurological (MRI) and nephrological data (kidney ultrasound [US], urinary and blood parameters) were evaluated.

## RESULTS

### Mutation screening strategy of known LCA genes

As a first step, 102 probands were subjected to LCA chip analysis (91 LCA and 11 EORD). In total, 30 sequence changes assigned as mutations by Asper Ophthalmics were identified in 47 individuals. Homozygous and compound heterozygous variants in one gene were each found in 13 patients; a single heterozygous variant was identified in 17 individuals. In addition, variants within two distinct genes were found in four patients. The zygosity of p.Glu1330X (*CRB1*) could not be determined in LCA-36. Confirmation of each mutation through direct sequencing identified two inconsistencies. At first, LCA-58 was genotyped heterozygously for the *AIPL1* mutation p.Trp278X by chip, while she was in fact homozygous. Secondly, a heterozygous p.Arg38AlafsX3 mutation in *AIPL1* (LCA chip version 2006) could not be confirmed in LCA-23. Instead, a heterozygous c.111C>T (p.=) variant was identified on the same nucleotide position. This miscall has previously been described (Henderson et al., 2007). In addition, subsequent sequencing of *GUCY2D* in LCA-51 revealed a heterozygous c.389del mutation that was not detected on the LCA chip. The variants c.2101C>T (p.Pro701Ser) (*GUCY2D*), c.3341A>G (p.Asp1114Gly) (*RPGRIP1*) (Vallespin et al., 2007a), c.286G>A p.Val96Ile (*AIPL1*) (Yzer et al., 2006) and C.1301C>T (p.Ala434Val) (*RPE65*) (Morimura et al., 1998) have already been reported as polymorphisms and were therefore discarded as mutations. Moreover, identification of the *GUCY2D* p.Pro701Ser variant in a homozygous state in both healthy parents from an LCA patient further supported its non-pathogenic nature. After the exclusion of these polymorphisms, variants were assigned to be mutations in 45 patients (39 LCA and 6 EORD).

Secondly, all patients with a heterozygous mutation identified through chip analysis were subjected to screening of the relevant gene. In addition, all patients with negative chip results underwent sequencing of 6 LCA genes. In the following sections, the molecular results are discussed in detail for each of the genes.

#### CEP290

*CEP290* was found to be the most frequently mutated gene in our cohort, accounting for 30% (27/91) of cases with isolated LCA (Table 1). Since the LCA chip did not contain *CEP290* variants at the onset of this study, only a fraction of currently known mutations were detected using this technique. The c.2991+1655A>G, c.4723A>T (p.Lys1575X) and c.3310-1_3310delinsAA mutations were the most recurrent, with gene-specific allele frequencies of 49%, 11% and 6%, respectively. Similar to previous studies, most of the mutations are either nonsense, frameshift or splice site mutations. Only two missense variants were identified, of which the pathogenic effect is currently uncertain (p.Ala1566Pro and p.Leu1694Pro) (Supp. Table S2). Overall, 13 novel *CEP290* mutations were identified to cause LCA. The complex allele c.3310-1_3310delinsAA has a predicted effect on splicing, which was confirmed by cDNA analysis (data not shown). The silent c.1824G>A change affects the last nucleotide of exon 18 and was also predicted to alter splicing (data not shown).

**Table 1:** Mutations identified in 80 unrelated patients with LCA/EORD, using LCA chip analysis and direct sequencing of *CEP290, CRB1, RPE65, AIPL1, GUCY2D* and CRX

				Allele 1				Allele 2		Reference
						
Patient	Origin	Par cons	Segr	Intron/exon	Nucleotide change	Amino acid change	Intron/exon	Nucleotide change	Amino acid change	
***CEP290***										

LCA-1	Belgium	-	X	I26	c.2991+1655A>G*	p.Cys998X*	I26	c.2991+1655A>G*	p.Cys998X*	(den Hollander et al., 2006)

LCA-2	Belgium	-	X	I26	c.2991+1655A>G	p.Cys998X	I26	c.2991+1655A>G	p.Cys998X	(den Hollander et al., 2006)

LCA-3+	Belgium	-	X	I26	c.2991+1655A>G*	p.Cys998X*	**E6**	**c.322C>T**	**p.Arg108X**	(den Hollander et al., 2006)

LCA-4	Belgium	-	NA	I26	c.2991+1655A>G	p.Cys998X	**E25**	**c.2695C>T**	**p.Gln899X**	(den Hollander et al., 2006)

LCA-5	Belgium	-	NA	I26	c.2991+1655A>G	p.Cys998X	E34	c.4393C>T	p.Arg1465X	(den Hollander et al., 2006), (Brancati et al., 2007) (CORS)

LCA-6	Belgium	-	X	I26	c.2991+1655A>G	p.Cys998X	E36	c.4723A>T	p.Lys1575X	(den Hollander et al., 2006), (Brancati et al., 2007; Perrault et al., 2007)

LCA-7^F^	Belgium	-	NA	I26	c.2991+1655A>G	p.Cys998X	E36	c.4723A>T	p.Lys1575X	(den Hollander et al., 2006), (Brancati et al., 2007; Perrault et al., 2007)

LCA-8	Belgium	-	NA	I26	c.2991+1655A>G	p.Cys998X	E36	c.4723A>T	p.Lys1575X	(den Hollander et al., 2006), (Brancati et al., 2007; Perrault et al., 2007)

LCA-9	Belgium	-	X	I26	c.2991+1655A>G	p.Cys998X	**E39**	**c.5344C>T**	**p.Arg1782X**	(den Hollander et al., 2006)

LCA-10	Lithuania	-	X	I26	c.2991+1655A>G	p.Cys998X	**E6**	**c.384 385del**	**p.Asp128GlufsX17**	(den Hollander et al., 2006)

LCA-11	Belgium	-	X	I26	c.2991+1655A>G	p.Cys998X	**E6**	**c.437del**	**p.Glu146GlyfsX17**	(den Hollander et al., 2006)

LCA-12	The Netherlands	-	NA	126	c.2991+1655A>G*	p.Cys998X*	E19	c.1859_1862del	p.Arg621IlefsX2	(den Hollander et al., 2006), (Perrault et al., 2007)

LCA-13	Belgium	-	NA	I26	c.2991+1655A>G	p.Cys998X	**E29**	**c.3422dup**	**p.Leu1141PhefsX5**	(den Hollander et al., 2006)

LCA-14	Belgium/Morocco	-	X	I26	c.2991+1655A>G*	p.Cys998X*	**E31**	**c.4001del**	**p.Thr1334IlefsX2**	(den Hollander et al., 2006)

LCA-15 (Perrault et al., 2007)	Belgium	-	NA	I26	c.2991+1655A>G	p.Cys998X	E37	c.4962_4963del	p.Glu1656AsnfsX3	(den Hollander et al., 2006), (Perrault et al., 2007)

LCA-16+	Belgium/Greece	-	X	I26	c.2991+1655A>G	p.Cys998X	E40	c.5493del	p.Ala1832ProfsX19	(den Hollander et al., 2006), (Brancati et al., 2007; Frank et al., 2008) (CORS)

LCA-17	Belgium	-	X	I26	c.2991+1655A>G	p.Cys998X	**E40**	**c.5519 5537del**	**p.Lys1840ArgfsX5**	(den Hollander et al., 2006)

LCA-18	Belgium	-	X	I26	c.2991+1655A>G	p.Cys998X	**E43**	**c.5865 5867delins GG**	**p.Glu1956GlyfsX9**	(den Hollander et al., 2006)

LCA-19	Belgium	-	X	I26	c.2991+1655A>G	p.Cys998X	**I13**	**c.1189+1G>A**	**Splice defect**	(den Hollander et al., 2006)

LCA-20+ (Yzer et al., 2006)	Belgium	-	NA	I26	c.2991+1655A>G	p.Cys998X	**121**	**c.2218-2A>C**	**Splice defect**	(den Hollander et al., 2006)

LCA-21	Belgium	-	X	I26	c.2991+1655A>G	p.Cys998X	**I28-E29**	**c.3310-1 3310delinsAA**	**Splice defect**	(den Hollander et al., 2006)

LCA-22	Belgium	-	NA	I26	c.2991+1655A>G	p.Cys998X	**I28-E29**	**c.3310-1 3310delinsAA**	**Splice defect**	(den Hollander et al., 2006)

LCA-23	Belgium	-	NA	I26	c.2991+1655A>G	p.Cys998X	**I28-E29**	**c.3310-1 3310delinsAA**	**Splice defect**	(den Hollander et al., 2006)

LCA-24	Belgium	-	NA	E36	c.4723A>T	p.Lys1575X	E36	c.4723A>T	p.Lys1575X	(Perrault et al., 2007)

LCA-25^F^	Belgium	-	X	E36	c.4723A>T	p.Lys1575X	**E35**	**c.4696G>C**	**p.Ala1566Pro UV**	(Perrault et al., 2007)

LCA-26	Belgium		X	**E18**	**c.1824G>A**	**p.=, splice site**	**E38**	**c.5081T>C**	**p.Leu1694Pro UV**	

LCA-27	Belgium	-	NA	I26	c.2991+1655A>G	p.Cys998X	?	?	?	(den Hollander et al., 2006)

SLS-1	Pakistan	FC	X	E2	c.21G>T	p.Trp7Cys	E2	c.21G>T	p.Trp7Cys	(Valente et al., 2006b) (CORS)

SLS-2	Belgium	-	NA	E36	c.4723A>T	p.Lys1575X	E34	c.4393C>T	p.Arg1465X	(Perrault et al., 2007), (Brancati et al., 2007) (CORS)

SLS-3	Belgium	-	NA	E36	c.4723A>T	p.Lys1575X	E34	c.4393C>T	p.Arg1465X	(Perrault et al., 2007), (Brancati et al., 2007) (CORS)

CORS-1+ (Brancati et al., 2007)	Belgium	SD	NA	E36	c.4723A>T	p.Lys1575X	E34	c.4393C>T	p.Arg1465X	(Perrault et al., 2007), (Brancati et al., 2007) (CORS)

LCA-JS-1	Belgium	-	X	I40	c.5587-1G>C	Splice defect	E31	c.3793C>T	p.Gln1265X	(Perrault 2007), (Baala et al., 2007) (ML)

LCA-JS-2 II-1				**E54**	**c.7366 7369del**	**p.Thr2457AlafsX27**	**E54**	**c.7366 7369del**	**p.Thr2457AlafsX27**	
				
LCA-JS-2 II-2	ND	+	X	**E54**	**c.7366 7369del**	**p.Thr2457AlafsX27**	**E54**	**c.7366 7369del**	**p.Thr2457AlafsX27**	

LCA-JS-3	Belgium	-	NA	**I28-E29**	**c.3310-1 3310delinsAA**	**Splice defect**	E54	c.7341dup	p.Leu2448ThrfsX8	(Sayer et al., 2006)

***CRB1***										

LCA-28	Belgium	-	NA	E7	c.2401A>T*	p.Lys801X*	E7	c.2401A>T*	p.Lys801X*	(den Hollander et al., 2001)

LCA-29 (Yzer et al., 2006)	Belgium	-	NA	E7	c.2401A>T*	p.Lys801X*	E5	c.1084C>T	p.Gln362X	(den Hollander et al., 2001), (Yzer et al., 2006)

LCA-30	Belgium	-	X	E7	c.2401A>T*	p.Lys801X*	E7	c.2290C>T*	p.Arg764Cys*	(den Hollander et al., 2001), (Lotery et al., 2001)

LCA-31 (Yzer et al., 2006)	Belgium	-	X	E7	c.2401A>T*	p.Lys801X*	E8	c.2688T>A*	p.Cys896X*	(den Hollander et al., 2001), (Hanein et al., 2004)

LCA-32 (Yzer et al., 2006)	Belgium	-	NA	E7	c.2401A>T*	p.Lys801X*	E8	c.2688T>A*	p.Cys896X*	(den Hollander et al., 2001), (Hanein et al., 2004)

LCA-33	Belgium	-	NA	E7	c.2401A>T*	p.Lys801X*	E9	c.2843G>A*	p.Cys948Tyr*	(den Hollander et al., 2001), (Lotery et al., 2001)

LCA-34	Belgium	-	X	E7	c.2401A>T*	p.Lys801X*	**I11**	**c.4006-1G>T**	**Splice defect**	(den Hollander et al., 2001)

LCA-35	Belgium	+	NA	E9	c.2843G>A*	p.Cys948Tyr*	E9	c.2843G>A*	p.Cys948Tyr*	(Lotery et al., 2001)

LCA-36	ND	-	NA	E9	c.2843G>A*	p.Cys948Tyr*	Ell	c.3988G>T*	p.Glu1330X*	(Lotery et al., 2001), (LCA chip)

LCA-37	Belgium	-	X	E9	c.2843G>A*	p.Cys948Tyr*	I8	c.2842+5G>A*	Splice defect*	(Lotery et al., 2001), (den Hollander et al., 1999)

LCA-38	Belgium	-	X	E9	c.2843G>A*	p.Cys948Tyr*	I8	c.2842+5G>A*	Splice defect*	(Lotery et al., 2001), (den Hollander et al., 1999)

LCA-39a				E9	c.2843G>A*	p.Cys948Tyr*	I8	c.2842+5G+A*	Splice defect*	(Lotery et al., 2001), (den Hollander et al., 1999)
				
LCA-39b	Belgium	-	X	I11	c.4005+1G>A	Splice defect	I8	c.2842+5G>A	Splice defect	(Hanein et al., 2004), (den Hollander et al., 1999)

LCA-40	Belgium	-	NA	I11	c.4005+1G>A*	Splice defect*	I8	c.2842+5G>A*	Splice defect*	(Hanein et al., 2004), (den Hollander et al., 1999)

LCA-41 II-1				**E7**	**c.2441 2442del**	**p.Leu814ArgfsX23**	**E9**	**c.3713 3716dup**	**p.Cys1240ProfsX24**	
				
LCA-41 II-2				**E7**	**c.2441 2442del**	**p.Leu814ArgfsX23**	**E9**	**c.3713 3716dup**	**p.Cys1240ProfsX24**	

LCA-42	ND	+	NA	E11	c.3879G>A*	p.Trp1293X*	E11	c.3879G>A*	p.Trp1293X*	(Hanein et al., 2004)

EORD-1 II-1				E9	c.2843G>A*	p.Cys948Tyr*	E7	c.2401A>T*	p.Lys801X*	(Lotery et al., 2001), (den Hollander et al., 2001)
				
EORD-1 II-2	Belgium	-	X	E9	c.2843G>A	p.Cys948Tyr	E7	c.2401A>T	p.Lys801X	(Lotery et al., 2001), (den Hollander et al., 2001)

EORD-2	Belgium		NA	E9	c.2843G>A	p.Cys948Tyr	**E4**	**c.929G>A**	**p.Cys310Tyr**	(Lotery et al., 2001)

EORD-3	Belgium	-	X	E9	c.2843G>A*	p.Cys948Tyr*	**E6**	**c.1472A>T**	**p.Asp491Val UV**	(Lotery et al., 2001)

EORD-4	Belgium		NA	E5	c.1084C>T*	p.Gln362X*	E5	c.1084C>T*	p.Gln362X*	(Yzer et al., 2006)

EORD-5	Belgium	-	NA	E7	c.2290C>T*	p.Arg764Cys*	E7	c.2290C>T*	p.Arg764Cys*	(Lotery et al., 2001)

***RPE65***										

LCA-43	Turkey	FC	X	E3	c.131G>A*	p.Arg44Gln*	E3	c.131G>A*	p.Arg44Gln*	(Simovich et al., 2001)

LCA-44	Turkey	FC	X	**E6**	**c.542C>T**	**p.Pro181Leu**	**E6**	**c.542C>T**	**p.Pro181Leu**	

LCA-45a				E7	c.700C>T*	p.Arg234X*	**E9**	**c.991 993dup**	**p.Trp331dup**	(Marlhens et al., 1997)
				
LCA-45b				**E9**	**c.991 993dup**	**p.Trp331dup**	**E9**	**c.991 993dup**	**p.Trp331dup**	

LCA-46	Portugal		X (c.10 22T> C)	E10	c.1022T>C*	p.Leu341Ser*	**E5**	**c.361delT**	**p.Ser121LeufsX6 *de novo***	(Morimura et al., 1998) (ARRP)

LCA-47	Belgium	-	X	E14	c.1590del*	p.Phe530LeufsX40*	E14	c.1590del*	p.Phe530LeufsX40*	(Yzer et al., 2006)

LCA-48	Belgium	-	X	E14	c.1590del	p.Phe530LeufsX40	E5	c.370C>T*	p.Arg124X*	(Yzer et al., 2006), (Morimura et al., 1998)

LCA-49	Belgium	-	X	E14	c.1590del*	p.Phe530LeufsX40*	11	c.11+5G>A*	Splice defect*	(Yzer et al., 2006), (Gu et al., 1997)

LCA-50	Belgium/Russia (mother)	-	X	**E9**	**c.886dupA**	**p.Arg296LysfsX7**	11	c.11+5G>A*	Splice defect*	(Gu et al., 1997)

***GUCY2D***										

LCA-51	Morocco/Belgium	-	NA	E2	c.389del	p.Pro130LeufsX36	**I13**	**c.2577-2A>C**	**Splice defect**	(Perrault et al., 1996)

LCA-52	Turkey	TC	NA	E8	c.1694T>C*	p.Phe565Ser*	E8	c.1694T>C*	p.Phe565Ser*	(Perrault et al., 1996)

LCA-53	Belgium		NA	E12	c.2302C>T*	p.Arg768Trp*	E12	c.2302C>T*	p.Arg768Trp*	(Lotery et al., 2000)

LCA-54	Morocco/Belgium	-	X	E12	c.2302C>T*	p.Arg768Trp*	E8	c.1694T>C*	p.Phe565Ser*	(Lotery et al., 2000), (Perrault et al., 1996)

LCA-55	Belgium	-	X	E12	c.2302C>T*	p.Arg768Trp*	**E14**	**c.2598G>C**	**p.Lys866Asn**	(Lotery et al., 2000)

LCA-56	Belgium/France	-	X	**E2**	**c.587A>T**	**p.Glu196Val UV**	**Ell**	**c.2132C>T**	**p.Pro711Leu UV**	

LCA-57	Africa	-	NA	E8	c.1724C>T*	p.Pro575Leu* UV	?	?	?	(Koenekoop et al., 2002)

***AIPL1***										

LCA-58 (Yzer et al., 2006)	Belgium	-	X	E6	c.834G>A*	p.Trp278X*	E6	c.834G>A	p.Trp278X	(Sohocki et al., 2000a)

LCA-59	Belgium	-	NA	E6	c.834G>A*	p.Trp278X*	E6	c.834G>A*	p.Trp278X*	(Sohocki et al., 2000a)

LCA-60	Belgium		NA	E6	c.834G>A*	p.Trp278X*	E6	c.834G>A*	p.Trp278X*	(Sohocki et al., 2000a)

LCA-61	Belgium	-	X	E6	c.834G>A*	p.Trp278X*	E6	c.834G>A*	p.Trp278X*	(Sohocki et al., 2000a)

LCA-62	Africa	-	*in cis*	E3	c.341C>T*	p.Thr114Ile*UV	?	?	?	(Sohocki et al., 2000b)
				c.1126C>T	p.Pro376SerUV					

***CRX***										

LCA-63	Belgium	SC	NA	E4	c.425A>G*	p.Tyr142Cys* UV	?	?	?	(Vallespin et al., 2007a)

LCA-64	Ruanda		NA	E3	c.724G>A*	p.Val242Met* UV	?	?	?	(Swain et al., 1997)

***RDH12***										

EORD-6	Belgium	-	X	E6	c.806_810del*	p.Ala269GlyfsX2*	E8	c.698T>A	p.Val233Asp	(Janecke et al., 2004), https://www.carverlab.org/carver-mutation-database

EORD-7	Belgium	+	X	E6	c.806_810del*	p.Ala269GlyfsX2*	**E7**	**c.524C>T**	**p.Ser175Leu**	(Janecke et al., 2004)

EORD-8	Belgium			E6	c.806_810del	p.Ala269GlyfsX2	E6	c.806_810del	p.Ala269GlyfsX2	(Janecke et al., 2004)

***RPGRIP1***										

LCA-65	Belgium			E16	c.2668C>T*	p.Arg890X*	?	?	?	(Gerber et al., 2001)

Novel mutations are indicated in bold.

+ patients carrying a heterozygous mutation in an additional gene: LCA-3 (*AHI1*, c.2273A>C, p.His758Pro), LCA-16 (*RPE65*, c.253C>T, p.Arg85Cys) (Stone, 2007), LCA-20 (*CRB1*, c.2401A>T, p.Lys801X) (den Hollander et al., 2001) and CORS-1 (*AHI1*, c.2433T>G, p.Asn81 1Lys).

* identified through LCA chip analysis.

F LCA-7 and LCA-25 are distantly related. X: segregation analysis performed and segregation confirmed. NA: no material available of family members. Reference: first publication describing the mutation in patients with LCA or EORD. In case of *CEP290*, these references may also refer to papers dealing with other phenotypes (phenotype mentioned between brackets). Seven patients were already described (corresponding reference is indicated in the first column).

Abbreviations used: par cons: parental consanguinity; segr: segregation; FC: first cousins; SC: second cousins; TC: third cousins; SD: second degree; ND: no data; UV, unclassified variant; LCA, Leber Congenital Amaurosis; SLS, Senior-Loken syndrome; JS, joubert syndrome; ARRP, autosomal recessive retinitis pigmentosa; CORS, cerebello-oculo-renal syndrome; ML, Meckel-like syndrome.

Since *CEP290* mutations may cause a phenotypic spectrum ranging from isolated LCA to more complex disorders, we analysed 13 additional probands suffering from LCA-JS, SLS or CORS. *CEP290* harbored mutations in seven of them (Table 1). Six probands carried known mutations, whereas a novel p.Thr2457AlafsX27 mutation segregated in family LCA-JS-2.

Sequencing of the entire coding region did not reveal a second mutation in LCA-27, while the pathogenic effect of one variant was uncertain in LCA-25 and LCA-26. Subsequent cDNA screening in LCA-25 and LCA-27 was normal, thereby making deep intronic splice site mutations or large exon deletions/duplications very unlikely. No RNA was available for LCA-26.

#### CRB1

Mutations in *CRB1* were found in 15 families with LCA (16%) and 5 families with EORD (Table 1). The LCA chip allowed the identification of a homozygous or compound heterozygous *CRB1* mutation in 15 probands (12 LCA and 3 EORD), and a heterozygous *CRB1* mutation in 3 patients. Sequencing of the whole coding region of *CRB1* in the latter revealed a known and novel mutation, respectively (LCA-29, p.Gln362X and LCA-34, c.4006-1G>T), and a novel unclassified variant (EORD-3, p.Asp491Val) on the second allele (Supp. Table S2). Sequencing of the total coding region identified compound heterozygous mutations in two additional probands. In one of them, *CRB1* screening was exceptionally performed without prior LCA chip analysis, given clear clinical indications for a *CRB1*-related phenotype (EORD-2). Indeed, this patient was compound heterozygous for the known p.Cys948Tyr mutation, and the novel p.Cys310Tyr variant, which is predicted to disrupt a disulfide bridge (Supp. Table S2). In addition, two sisters with LCA carried two novel frameshift mutations (LCA-41). As previously described, the p.Lys801X and p.Cys948Tyr mutations were most frequent, showing gene-specific allele frequencies in the LCA cohort of 27% and 23%, respectively.

#### RPE65

Eight cases with LCA showed mutations in *RPE65* (9%) (Table 1). LCA chip analysis identified a homozygous *RPE65* mutation in LCA-43 and LCA-47 and two compound heterozygous mutations in LCA-49. In addition, a heterozygous mutation was detected in four patients through LCA chip analysis. Sequencing of *RPE65* in these individuals identified three novel mutations and the known p.Phe530LeufsX40 mutation which was not yet present on the LCA chip at the time (LCA-48). Although the evidence for a pathogenic nature of the novel mutation p.Trp331dup is not conclusive, segregation in patient LCA45a, her affected aunt LCA45b and her (healthy) parents sustains a causal role. The two other novel mutations result in a frameshift (LCA-46 and LCA-50). Following sequencing of the total coding region, one additional proband was found to be homozygous for the novel *RPE65* mutation p.Pro181Leu (LCA-44) (Supp. Table S2). Interestingly, segregation analysis of the mutations found in LCA-46 could only confirm segregation of p.Leu341Ser in the mother, suggesting that p.Ser121LeufsX6 occurred *de novo* (paternity confirmed).

#### GUCY2D

Mutations in *GUCY2D* were found in seven probands with LCA (8%) (Table 1). Five of them were identified with *GUCY2D* mutations using the LCA chip. One was homozygous for p.Phe565Ser (LCA-52), while three others carried p.Arg768Trp. One of the latter was homozygous (LCA-53); the other two were compound heterozygous for p.Phe565Ser (LCA-54) and the novel missense change p.Lys866Asn (LCA-55), respectively (Supp. Table S2). In addition, LCA-57 was heterozygous for the p.Pro575Leu variant that was previously identified in the mother of an LCA patient (Koenekoop et al., 2002). However, no second mutation was found. LCA-56 was compound heterozygous for the novel missense changes p.Glu196Val and p.Pro71 1Leu (Supp. Table S2). In addition, a novel splice site mutation was identified in LCA-51 (c.2577-2A>C).

#### AIPL1

Only two distinct *AIPL1* variants were detected through LCA chip analysis in five LCA patients (5%) (Table 1). The p.Trp278X mutation occurred homozygously in four probands (LCA-58 to LCA-61). In addition, a heterozygous p.Thr1 14Ile variant was found in proband LCA-62. Direct sequencing of the *AIPL1* gene identified the known variant p.Pro376Ser (missing signal on LCA chip). Segregation analysis in the parents, however, revealed a *cis*-allelic inheritance from the mother. No further mutations were detected following additional sequencing of *AIPL1* in other patients.

#### CRX

LCA chip analysis identified 2 *CRX* missense variants in two LCA patients (Table 1). The p.Tyr142Cys variant was previously described as a mutation (Vallespin et al., 2007a). Stone and colleagues, however, considered this variant as a polymorphism based on the estimate of pathogenic probability and the identification of this variant in a patient with two disease-causing alleles in another LCA gene (Stone, 2007) (LCA-63). The pathogenicity of the second variant p.Val242Met also remains unclear (LCA-64) (Swain et al., 1997; Rivolta et al., 2001; Chen et al., 2002). Given their uncertain pathogenic potential, both variants were discarded as mutations for further calculations.

#### RDH12

Mutation screening of *RDH12* was performed downstream of LCA chip results involving this gene. A heterozygous p.Ala269GlyfsX2 mutation was identified in two probands with EORD. Subsequent sequencing of *RDH12* identified an additional missense change in both patients, p.Val233Asp (EORD-6) and p.Ser175Leu (EORD-7) respectively (Supp. Table S2). According to UniProt, the Ser175 residue might be a substrate binding site (http://www.uniprot.org/uniprot/Q96NR8). A known mutation located in the same codon, p.Ser175Pro, lacks the ability to catalyze the reduction of retinaldehyde to retinol *in vitro* (Lee et al., 2007). In addition, a homozygous p.Ala269GlyfsX2 mutation was identified in patient EORD-8, for which *RDH12* sequencing was performed prior to LCA-chip analysis (upon request).

#### RPGRIP1

Similarly, screening of *RPGRIP1* was performed in the context of LCA chip analysis. In one LCA patient, a heterozygous mutation was identified. Sequencing of *RPGRIP1*, however, did not identify a second mutation (LCA-65).

#### Mutations in multiple LCA genes

For the assessment of the potential involvement of a second gene in LCA, only variants with significant pathogenic potential were taken into account (see above). Two patients with *CEP290*-related LCA displayed a heterozygous mutation in another LCA gene: LCA-16 was heterozygous for the known p.Arg85Cys mutation in *RPE65*, while LCA-20 carried the common p.Lys801X mutation in *CRB1*.

### Identification of potential modifier alleles in the *AHI1* gene

The *AHI1* gene was sequenced as a candidate modifier gene in eight patients with *CEP290*-related LCA who presented with mental retardation. Four of them were diagnosed with LCA (LCA-3, LCA-20, LCA-23 and LCA-24); two patients also suffered from NPHP (SLS-2 and SLS-3) and in two other cases, the LCA phenotype was part of a JS diagnosis (CORS-1 and LCA-JS-3). A MTS was absent on brain imaging in two isolated patients with LCA (no data were available for LCA-20, LCA-24, SLS-2 and SLS-3).

A heterozygous novel *AHI1* p.Asn811Lys mutation was found in the most severely affected patient CORS-1, out of three patients with the same *CEP290* genotype but different neurological involvement (SLS-2, SLS-3 and CORS-1). Moreover, *AHI1* screening in the five remaining patients revealed a second heterozygous missense variant, p.His758Pro, in LCA-3. Conservation and *in silico* predictions for both changes suggest a possible effect on protein structure/function (Supp. Table S2). Interestingly, exonic splicing enhancer (ESE) predictions point to a change in ESEs for both variants (data not shown). Moreover, both changes are located in a conserved WD-40 repeat (http://www.uniprot.org/uniprot/Q8N157) and were absent in > 340 Belgian control individuals.

In addition, SLS-2 was found to be heterozygous for the known p.Ser1123Phe change. Although it concerns a potentially pathogenic variant that affects a phosphorylation site and is located in a highly conserved region (Dephoure et al., 2008), this change was considered a polymorphism because of its frequency in the Dutch population and the observation that it did not segregate in a family with JS (Valente et al., 2006a; Kroes et al., 2008).

### Clinical findings

**Figure 1:**
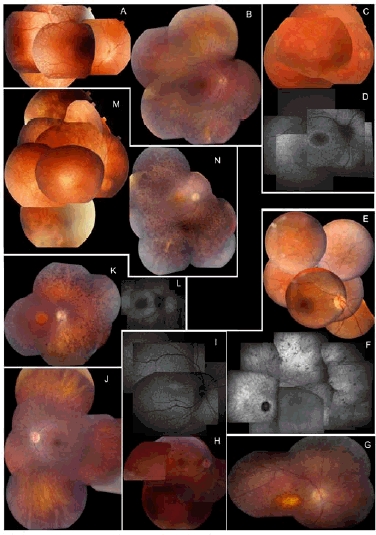
Clinical characteristics of eight LCA patients with an established molecular diagnosis, illustrating characteristic phenotypic features associated with different genotypes. *CEP290*. **A & B**: Early and later stage phenotype in right eye (RE) in LCA-3 at age 3 and 18 years respectively; note marbleized aspect of midperiphery at age 3, evolving towards atrophy later; macula stays well-preserved throughout evolution. **C & D**: Fundus and autofluorescence (AF) image of RE of LCA-25 at age eight years; note concentric hyperautofluorescent ring around macula suggesting a watershed zone between better and more affected retina with probably central area the better; midperipheral retina shows diffuse mottled hyperautofluorescence suggesting widespread outer retinal disease. **E & F**: Fundus image of RE and infrared image of left eye (LE) of LCA-7 at age 33 and 49 years respectively; note pigment epithelium alterations in the mid- and far periphery of retina but no intraretinal pigmentation, and with fair preservation of macular area at age 33; at age 49 macula is still fairly well-preserved, but outer retinal atrophy and spicular intraretinal pigmentation is now prominent. *CRB1*.**G**: Fundus of RE of LCA-3 9a at age 16, showing typical yellowish discoloration of atrophic macula, surrounded by nummular type of intraretinal pigmentation; mild pseudopapilledema and prepapillary paravascular fibrosis also visible, as is peripheral greyish hue of outer retinal atrophy with fine white flecks and nummular pigmentation. *GUCY2D*. **H & I**: Fundus and AF image of RE of LCA-55 at age 9 years; fundus is essentially quite normal with only mild pigment epithelium alterations in the retinal periphery; however, AF image shows hyperautofluorescence in central macular area. *RPE65*. **J**: Fundus of LE of LCA-49 at age 10 who subsequently underwent gene therapy with AAV2-hRPE65v2 in RE (Maguire et al. 2009); apart from some discrete pigment epithelium alterations fundus is essentially normal; autofluorescence imaging could not be obtained due to lack of lipofuscin accumulation in retinal pigment epithelium (RPE) typical of this type of LCA. *AIPL1*. **K & L**: Fundus and AF image of RE of LCA-61 at age 19 years; central macular atrophy with yellowish hue is surrounded by area of better preserved peripheral macula; outer retinal atrophy with spicular intraretinal pigmentation visible in periphery; AF shows black area of atrophic central macula, but is typically not surrounded by hyperautofluorescent ring. *RDH12*. **M & N**: Fundus of EORD-7 at age 5 and 19 years respectively; note mild macular RPE changes which become more prominent with age; mild predominantly spicular intraretinal pigmentation also increases with age; however, preservation of patches of normal peripheral retina are most striking feature; these patches remain over time.

**Figure 2:**
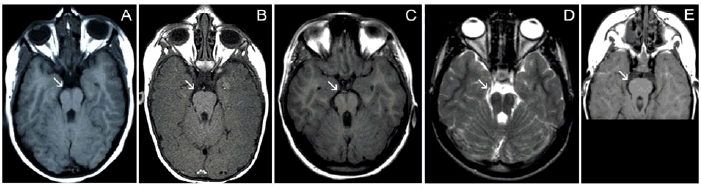
Magnetic resonance imaging (MRI) showing characteristic molar tooth sign (MTS) in five patients with Joubert syndrome/cerebello-oculo-renal syndrome due to mutations in *CEP290* (all are axial sections through midbrain). Images organized from left to right; arrows indicate MTS of midbrain present in all due to midbrain malformation with hypoplastic cerebellar vermis and midline cleft (all images are T1 weighted except for panel D which is T2 weighted). A) CORS-1 at age 5 years; B) LCA-JS-1 at age 7 years; C) LCA-JS-2 II-1 at age 14 years; D) LCA-JS-2 II-2 at age 17 years and E) LCA-JS-3 at age 2 years.

Extensive ophthalmological data (best corrected visual acuity [BCVA], refraction, ERG, visual fields, color vision testing, fundus aspect both with white light and autofluorescence imaging and the presence of nystagmus, night blindness, photophobia and additional features) as well as associated manifestations in patients with *CRB1, RPE65, GUCY2D, AIPL1, CRX, RPGRIP1* and *RDH12* mutations are summarized in detail in Supp. Table S3. In addition, Figure 1 depicts several representative fundi from patients with an established molecular diagnosis. An MTS due to midbrain abnormalities with cerebellar vermis aplasia was demonstrated in five patients with JS-LCA/CORS and *CEP290* mutations (Figure 2).

Special attention was paid to the *CEP290*-related ocular phenotype, since this has been described in only a few LCA studies so far. It appeared that this phenotype displays only limited fundus alterations in the first few years of life. In a small subset of patients, no fundus abnormalities were obvious early on, while in the majority a marbleized fundus and/or salt and pepper aspect was seen during the first decade. This aspect further evolved from young adulthood into progressive outer retinal atrophy in the midperiphery with relative sparing of the central macula. Abnormalities of the central macula were absent in our patient cohort, despite the impression that *CEP290*-related disease is probably of a cone-rod type (based on ERG findings and the occurrence of photophobia). Of note is the presence of a hyperautofluorescent ring around the central macula on AF imaging, observed in four patients starting from the age of six (LCA-2, LCA-3, LCA-7 and LCA-25). Mild intraretinal spicular pigment migration occurred in three patients at an age between 7 and 33 years old (LCA-JS-1, LCA-6 and LCA-7). This aspect became even more pronounced at the age of 49 in patient LCA-7, where a predominant spicular pigmentation was mixed with less frequent intraretinal pigment migration with a nummular aspect. Visual acuity of this group was mostly limited to light perception. In the few patients with better preserved central vision, basic color vision was present and visual fields varied from severely concentrically constricted (LCA-8) to sparing of the central 30° at an age of 49 (LCA-7) (Supp. Table S3).

## DISCUSSION

### Genotypes of the Belgian LCA population

In a cohort of 91 unrelated LCA patients, mainly originating from Belgium, a total of 61 different mutations (including 9 UVs) were found in 7 genes. Homozygous or compound heterozygous mutations were detected in 65% (59/91) of probands, whereas only one heterozygous mutation could be identified in 4% (4/91) of probands. In addition to isolated LCA, this study also identified mutations in eight probands with EORD and seven probands with syndromic LCA (SLS, LCA-JS and CORS) (Table 1).

LCA chip analysis proved to be a powerful initial tool as mutations were found in 41% (37/91) of patients with LCA. Subsequent sequencing of 6 genes (*CEP290, CRB1, RPE65, GUCY2D, AIPL1* and *CRX*) enabled us to identify mutations in an additional 28% of cases. Of note, the majority of mutations found in the latter probands are now included in the LCA chip, increasing its detection rate to 65% if our cohort would have been analyzed using the latest version (v8, 641 variants).

Segregation of disease alleles was demonstrated in 41 out of 43 families available, with two exceptions. The first one is LCA-62, in which the *AIPL1* variants p.Thr1 14Ile and p.Pro376Ser were located in *cis* on the maternal chromosome. This finding challenges a previous study proposing this genotype as causal in an LCA patient (Sohocki et al., 2000b). In the second case, only one of two mutations identified in LCA-46 was found in the mother but not in father, suggesting that the other mutation arose *de novo* (*RPE65*, p.Ser121LeufsX6, nonp-aternity excluded) (Table 1). Of note, two different mutations in *CEP290* were identified in CORS-1, originating from a consanguineous marriage, which illustrates that assuming homozygosity in offspring from a consanguinous mating can be a potential pitfall for the identification of the causal defect (Table 1).

Our data demonstrate a key role for *CEP290* in the Belgian LCA population, as *CEP290* mutations were identified in 27 probands (30%). Although the prevalence of *CEP290* mutations is not this high worldwide (Simonelli et al., 2007; Vallespin et al., 2007b; Seong et al., 2008; Li et al., 2009; Sundaresan et al., 2009), this study corroborates the importance of *CEP290* in the Northwestern European population (den Hollander et al., 2006; Perrault et al., 2007). The second most frequently mutated gene in our LCA population was *CRB1* (16%), followed by *RPE65* (9%), *GUCY2D* (8%) and *AIPL1* (5%), which is in agreement with previous data (den Hollander et al., 2008). The *RPGRIP1* gene – in this study only investigated with the LCA chip – accounted for less than 1% of the LCA population.

In total, 30 novel mutations/variants were identified in this study (Table 1, Supp. Table S2). Interestingly, 5 out of 16 novel mutations in *CEP290* are located nearby known changes: c.384_385del (c.384_387del, c.381_382delinsT) (Baala et al., 2007; Perrault et al., 2007), c.2218-2A>C (c.2218-4_2222del and c.2218-15_2220del) (Sayer et al., 2006; Stone, 2007), c.3310-1_3310delinsAA (c.3310-1G>C) (Tory et al., 2007), c.5519_5537del (c.5515_5518del) (Sayer et al., 2006) and c.5865_5867delinsGG (c.5866G>T) (den Hollander et al., 2006). A similar observation was made for *RDH12*, in which the novel mutation p.Ser175Leu affects the same codon as the known mutation p.Ser175Pro (Perrault et al., 2004). Overall, these regions/codons might be more prone to mutational events.

For several genes, a limited number of recurrent mutations made up the majority of mutated alleles. This was certainly the case for *CEP290*, in which c.2991+1655A>G was found in 89% of all LCA patients with *CEP290-* related pathology. Together with p.Lys1575X and c.3310-1_3310delinsAA, a significant fraction of mutated alleles was identified in the LCA population (35/53 alleles). So far, p.Lys1575X has only been found in patients originating from northern France (Perrault et al., 2007) or Belgium (Brancati et al., 2007). This potential founder effect is supported by our study, since all patients who carry p.Lys1575X live in Flanders (northern part of Belgium). A similar regional prevalence was seen for p.Ala1832ProfsX19, which was inherited from the Greek father of LCA-16. The same mutation occurred in an Italian patient with CORS (Brancati et al., 2007) and in two consanguineous families of Kosovar-Albanian and Kosovar origin with MKS, sharing a common haplotype (Frank et al., 2008). For the other genes, the following mutations presented with a gene-specific allele-frequency of at least 20% in the LCA population: p.Lys801X (*CRB1*, 27%), p.Cys948Tyr (*CRB1*, 23%), p.Phe530LeufsX40 (*RPE65*, 25%), p.Arg768Trp (*GUCY2D*, 31%), p.Phe565Ser (*GUCY2D*, 23%) and p.Trp278X (*AIPL1*, 89%). The presence of all but one of these mutations on the LCA chip significantly contributed to its high detection rate.

In six LCA patients, only one mutation was found after sequencing of the gene apparently involved following chip testing. Notably, we might have failed to detect deep intronic and regulatory mutations or multi-exon deletions, as were recently demonstrated in *CEP290* (Travaglini et al., 2009). In addition, it cannot be excluded that the phenotype is caused by mutations in a different gene, as was the case for LCA-20. Furthermore, new mutations in the other known genes cannot be ruled out here. Their contribution is expected to be limited however, taking into account the high detection rate obtained using the current strategy. Finally, these patients may carry mutations in as yet unknown genes.

### Phenotypes of the Belgian LCA population

In addition, the phenotypes of patients with a molecular diagnosis were extensively studied. For all genes, nystagmus and hypermetropia were recurrent features. The oculodigital sign (plus enophthalmos) was often seen in all but *RPE65*-related LCA patients. A relatively higher incidence of both keratoconus and cataract was observed in the *CRB1*-related group, which may reflect secondary effects of a more severe retinal dystrophy compared to other genes. Indeed, several retinal abnormalities such as macular atrophy and intraretinal pigment migration already became apparent in the first decade of life in patients with *CRB1*-related disease, being earlier than generally seen for the other genes. In addition, a yellowish discoloration of the central macula was often observed. However, this feature is not entirely gene-specific, since it also occurred in a patient carrying *RDH12* mutations (EORD-7). This feature may be due to more severe outer retinal atrophy in the macula in *CRB1*- and *RDH12*-related disease, which may cause more intense scleral light reflection due to less absorption by the atrophic retinal pigment epithelium, with consequent highlighting of the macular luteal pigment.

The *RPE65*-related phenotype proved to be typically associated with a fundus appearance which is essentially normal during first years and displays only later on fundus alterations which are initially mild. Visual acuity is generally somewhat better than that seen in *CRB1* -related LCA. However, it seems to be the relatively slow evolution of the phenotype which makes it particularly suitable for therapeutic intervention (Bainbridge et al., 2008; Hauswirth et al., 2008; Maguire et al., 2008). For two patients from our cohort (LCA-47 and LCA-49), *RPE65* gene-replacement therapy resulted in better visual function (Maguire et al., 2009). In addition, two patients with *RPE65* mutations reported a period of increased visual function, possibly reflecting postnatal physiological cone maturation (LCA-44 and LCA-47) (Koenekoop et al., 2007). Hanein et al. classified both *CRB1*- and *RPE65*- related LCA as rod-cone dystrophies because of a predominant occurrence of night blindness (Hanein et al., 2004). All *RPE65*-related phenotypes in this study correspond to this classification. In the *CRB1*-group, however, six patients with LCA and two with EORD also suffered from photophobia, even before the onset of night blindness in EORD-3.

Similarly, the *GUCY2D*-related phenotype was found to have only limited fundus abnormalities. Although this phenotype was previously categorized as cone-rod dystrophy, one patient in our cohort had severe night blindness before photophobia became apparent (LCA-54).

In the patients with *AIPL1* mutations, an RP-like phenotype emerged by their teenage years at the latest, and a maculopathy with (partial) outer retinal atrophy was typically present in the majority of cases (Dharmaraj et al., 2004).

A unique feature of *RDH12*-related early-onset dystrophy was the occurrence of areas with complete preservation of the chorioretina in the retinal periphery, alternating with regions of total atrophy (EORD-7).

Notably, our study is one of the first reporting on the ocular phenotype of a larger group of LCA patients with *CEP290* mutations (Supp. Table S3). In keeping with previously reported findings (Perrault et al., 2007), it appeared to be that of a severe cone-rod type retinal dystrophy. Visual acuity was mostly limited to light perception, as recently described (Walia et al., 2010). Interestingly, a limited subset of patients displayed no obvious retinal abnormalities in the first years of life. In general, the fundus contained either small white dots or, more frequently, a marbleized or salt and pepper aspect in the first to second decade. In two patients aged 18 and 49, predominant spicular pigment migration was observed (LCA-6 and LCA-7), in contrast to the reported nummular pigmentation in one patient in the fourth decade (den Hollander et al., 2006). In one patient, nummular pigmentation was described in the first decade (LCA-10). Interestingly, a more severe phenotype was seen in LCA-20, who carried a heterozygous *CRB1* null allele on top of two mutations in *CEP290*.

### Extra-ocular features of *CEP290*-related LCA and potential modifier alleles in *AHI1*

Several patients with *CEP290*-related retinal dystrophy showed additional systemic features. Two patients with isolated LCA had several symptoms suggestive of renal dysfunction (LCA-3 and LCA-23, Supp. Table S3). LCA-3 suffered from growth retardation, polydipsia, enuresis nocturna and diurnal incontinence. Kidney US at the age of seven, however, was normal. In LCA-23, kidney US at the age of three revealed increased echogenicity and kidneys without clear cortico-medullar differentiation. Despite this observation, no clinical nephrological manifestations were present at the age of 17. Since the age of onset of end-stage renal disease caused by *CEP290* mutations may exceed the age of 20 (Helou et al., 2007; Tory et al., 2007), a close nephrological follow-up of these patients is required. Interestingly, both of these patients carry the recurrent c.2991+1655A>G mutation, which so far has only been reported in LCA patients without any other associated pathology. Of note, kidney US was available for only a subset of patients, and in general performed very early in life, when developing kidney disease might be difficult to detect.

In addition, four patients suffered from recurrent otitis media (OM) (LCA-5, SLS-1, SLS-3 and CORS-1, Supp. Table S3). Although this is common in childhood, it is worth mentioning that it is also a clinical manifestation often seen in primary ciliary dyskinesia (PCD), a genetically heterogeneous disorder of motile cilia (Leigh et al., 2009). In a few cases, PCD with OM was associated with X-linked RP, caused by mutations in *RPGR* (Shu et al., 2007). Notably, RPGR is a centrosomal protein that interacts with CEP290 (Chang et al., 2006). Moreover, loss-of-function experiments of *CEP290* in zebrafish caused developmental abnormalities of the otic cavity (Sayer et al., 2006).

Strikingly, 33% of patients with *CEP290*-related isolated LCA presented with mental retardation and/or autism, in contrast to only 8% of patients with mutations in the other genes. Subtle brain abnormalities such as broadened lateral ventricles were seen on MRI in some patients. Of note, brain imaging was not available for a subset of patients. Additional neurological manifestations included movement abnormalities (LCA-21), and dyspraxia and balance/coordination problems (LCA-18), the latter of which was also evident in two patients with syndromic *CEP290*-relatedLCA (SLS-2 and CORS-1) (Supp. Table S3).

Taken together, these extra-ocular manifestations fit well into the broad clinical spectrum of *CEP290* mutations, varying from isolated LCA to the lethal MKS. In addition, the *CEP290* allelic spectrum is highly complex. Mutations associated with isolated LCA in this study were previously reported in other ciliopathies (Table 1), albeit always in compound heterozygosity with a different mutation in the distinct phenotypes. Moreover, identical *CEP290* genotypes can display interfamilial variable expressivity and intrafamilial variation of the neurological phenotype was observed in several families with *CEP290*-related pathology (Coppieters et al., 2010). The complexity of *CEP290*-related disease is further illustrated by two cases from this study.

The first one is SLS-1, homozygous for p.Trp7Cys. Valente and coworkers identified the same mutation in patient with CORS, also of Pakistani origin (COR22, II:1). Despite a similar ocular and renal phenotype, both patients significantly differ in their neurological phenotype. A second and even more pronounced example is the variability in both nephrological and neurological involvement in three unrelated patients with the same p.Lys1575X/p.Arg1465X genotype (SLS-2, SLS-3 and CORS-1). Patients SLS-3 and CORS-1 displayed a similar clinical course of renal disease, with renal failure at the age of 16 and 14 years, respectively. In contrast, renal insufficiency in patient SLS-2 was not substantiated until the age of 30 (Supp. Table S3). Neurological signs of these three patients ranged from a mild mental handicap (SLS-2) over severe autism in combination with moderate mental retardation (SLS-3) to severe mental retardation associated with ataxia and a MTS (CORS-1). An MRI was not available for the other two patients, however.

The *AHI1* gene was screened as a candidate modifier gene in these three patients. Strikingly, CORS-1, with the most severe nephrologic and neurologic phenotype, carries a heterozygous novel p.Asn811Lys mutation in *AHI1*, which was absent in the two other patients. Upon screening of *AHI1* in five additional patients with *CEP290*-related disease and neurological involvement, a novel missense variant, p.His758Pro, was identified in LCA-3. Mutations in *AHI1* encoding Jouberin are responsible for JS, with retinal involvement in 75% and renal involvement in less than 10% of all *AHI1*-associated patients (Kroes et al., 2008). Interestingly, both p.Asn811Lys and p.His758Pro affect conserved residues and are located in the predicted WD40-repeat, a domain conserved across all eukaryotes, mediating functions such as vesicular trafficking (Li and Roberts, 2001). Since AHI1 and CEP290 appear to be in the same pathway through their interaction with rab8a, mutations in one of both genes may modify a phenotype caused by the other (Kim et al., 2008; Tsang et al., 2008; Hsiao et al., 2009). Tory and colleagues already suggested a similar potential epistatic effect of *CEP290* and *AHI1* mutations on phenotypes related to *NPHP1*, encoding another interactor of AHI1 (Tory et al., 2007; Eley et al., 2008). Strikingly, one of the *AHI1* variants they described as a potential modifier for neurological involvement in patients with *NPHP1* mutations, p.Arg830Trp, was recently identified as a modifier allele for retinal degeneration in patients with NPHP, independent of a primary *NPHP1* mutation (Louie et al., 2010). Of note, four out of seven patients in the study from Tory and coworkers carrying p.Arg830Trp displayed visual impairment, with one blind individual (Tory et al., 2007). The p.Arg830Trp variant might affect AHI1 complex stability/formation (Louie et al., 2010). The variants identified here, assumed to represent a neurological modifier in patients with LCA, might disrupt interactions with other proteins, thereby influencing AHI1 function in other organ systems.

Overall, a molecular diagnosis of *CEP290* mutations might have considerable consequences towards the clinical prognosis of an individual. Given the potential involvement of ciliary modifiers and the presence of cilia throughout the whole body, the development of various additional clinical manifestations should be taken into account. As for LCA, both children and (young) adults should have a long-term close clinical neurological and nephrological follow-up, since some features have a later onset.

In conclusion, molecular testing identified mutations in 69% of our LCA cohort, with a major involvement of *CEP290*. Detailed phenotyping of all patients with a molecular diagnosis revealed novel insights, mainly into the CEP290-related retinal phenotype, which is well documented for the first time in a larger patient group. The variable age-of-onset of the extra-ocular features emphasizes the importance of long-term clinical follow-up of LCA patients with *CEP290* mutations. Moreover, the identification of potential modifiers of *CEP290*-related disease might contribute to a refined prognosis based on a molecular diagnosis. Finally, our findings fit with previous observations suggesting that the phenotype of ciliopathies is most likely determined by the synergistic effect of all variants occurring in the ciliary proteome.

## Figures and Tables

**Supp. Table S1:** Exon-flanking primers for the amplification of the human *CEP290, CRB1, RPE65, GUCY2D, AIPL1, CRX, RDH12, RPGRIP1* and *AHI1* genes and for the c.2991+1655A>G mutation in *CEP290*

Gene	Exon	Forward primer (5′-3′)	Reverse primer (5′-3′)
	
	Exon 2	tttgtggcccaattgtctg	ccacctaagtaaacagaaaagcaac
	
	Exon 3	ccaaggtgcttaattggtca	tttcccctacacaccctttt
	
	Exon 4	tttactgaacgtctccatgtgc	tggcagatccataaaataggag
	
	Exon 5	ttctagactcctattttatggatctgc	ttcacaaccatatgctcagtcc
	
	Exon 6	atctgcactgaagtataatgc	tggtgatgacaaaatgaaca
	
	Exon 7	ggtgggagaattgcttgaac	acccgcatagacctgagatg
	
	Exon 8	ttggttctactgagccaaataatg	tctgaaggtaaccaaacacaaca
	
	Exon 9	ggtgaggctttaagtgtggtg	cttaatgaccaagacaggcaaa
	
	Exon 10	tggtcaatgccaattagtaaagg	gtgaggtgattggagaaacaca
	
	Exon 11	ttccaggatgacttcaatgataaa	aactcattgatgtgaaagaggtca
	
*CEP290*	Exon 12	cagagattatgccagtagttgctc	ttgggaccaggtggtagaag
	
	Exon 13	aaaaggcatacttgtacccaca	tccatcatttacaaatgtaagcac
	
	Exon 14	aatggcataccacttttcttgc	tggcaaaaagtaaatgctcaaag
	
	Exon 15	gcatatgtacattttcctttagac	actccaaccccataaaatct
	
	Exon 16	tcacagaaagttacctcattcttc	cctctttcttgagccatttg
	
	Exon 17	tgtaggccttgaaccaaagac	ttcaggactgaacccactgac
	
	Exon 18	ggatcacgaggtcagaagat	aaatgagaagcttgtattggct
	
	Exon 19	gggattaagatcaccatctgc	acagcaaggcaaatcaactg
	
	Exon 20	aactcaactattcacgttggattt	ttgaaagcagctcaagaaaaac
	
	Exon 21	tatcatgcttggcaatgaa	tcttataaggcacttattttccac
	
	Exon 22	ggcatttctaagcagaaactgaa	atctgcatgctttggtgatg
	
	Exon 23	tgtgttgcttacagatttggtga	caggataatcccaggcttaatg
	
	Exon 24	tgataactttgttgccttgcat	tccgaatccatctgaagagc
	
	Exon 25	gatcaagtccaacaagatgc	cagaagaagcaattatgacaaa
	
	Exon 26	tgcatgtttttcttacatgg	aaatgcaggcaaactttaat
	
	Intron 26	cattagaaagtcctaggcaagagacatc	agtaaggaggatgtaagactggagatagag
	
	Exon 27	ggacacagccaaaccatatc	caggattattcatctgcctaagtt
	
	Exon 28	tccatggaactataatgctttc	tctgctttccttttaaacaattc
	
	Exon 29	tttaccctccttcagtctgttc	ttaaccctgttaaaaccgatct
	
	Exon 30	gcaaacatgataacctctgatgg	ctgggcaacagaatgagacc
	
	Exon 31_1	agttccagctatgtttgcac	ctcgtttggagggaagaaac
	
	Exon 31_2	ccaagggtaaagctccacta	ttgtagatctcatgtgccact
	
	Exon 32	caggagaatggcatgaacc	atcattatcatcaatggaggaatgt
	
	Exon 33	tcacctctctgagtttgtatt	cagttgcagcattgagagtaa
	
	Exon 34	atcttgtttgttactctgtagcat	tggctattagaaacattataggag
	
	Exon35_1	aagcatgcaaataactgctgtc	accttgcttgcatgtttgc
	
	Exon35_2	aatcaatgaactgaggcttcg	ggtgtaatccaatcacatgcaa
	
	Exon 36	gaggggacatgcataccagt	cggtgagctacaggaggaag
	
	Exon 37	tttgatcatttgaggaaccaaa	ccagcagtcctgaggataaa
	
	Exon 38	gttgcagtcagccgagattg	actttttattacaacacggagat
	
	Exon 39	ttcaatgtggaaagaattgagtg	tgtctagccaccaacagtgc
	
	Exon 40_1	tcctcaaggtagacttgacatgaa	ttgccttttcagttcatcattc
	
	Exon 40_2	ctactacctctatattgtaaatcagaca	ttgtgggtgtttgttgaaga
	
	Exon 41	aaaatgcagaagcagctacca	ttcaatactgcttatagtcctcaa
	
	Exon 42	tcaacctgtatagcaaaatgaaca	cgagatcacaggaaaatcca
	
	Exon 43	tggattttcctgtgatctcg	tcaattcacatgggaaaagaaa
	
	Exon 44	catttaaaggaggccttcagtg	tgagaaagatgtaatgcttttgg
	
	Exon 45	ggctttcaccagaacactcc	ggccttcaacaaataaatgct
	
	Exon 46	tgcatcaggcaataatgtgg	cagatgcagaataaacactgaaa
	
	Exon 47_1	gctgcatgattttaggaatgtc	caatttttcattttcctgctca
	
	Exon 47_2	tggtagaagtggaaagacaatcc	cccttagccttgcctctcat
	
	Exon 48	aacgttgggaacttcgttct	tggtggaatgtgatgacagc
	
	Exon 49	aggaagaaaccaggttatcca	ttgaatacactgaatctatgagaaca
	
	Exon 50	ttgccaccactttttaatgc	ggggtgcccttcagttagat
	
	Exon 51	tgcttgtctctagttgtagca	ctaggacaatgccagttatgc
	
	Exon 52	cgtgaaggcttttgtattcca	aagacccaaagcttatcaggaa
	
	Exon 53	ggagggaggcagcattaagt	tgttaggaatagtcagatgaaca
	
	Exon 54	tgcctttattgctgtatttgacc	tcggagaactgcttatttcca

	Exon 1	atgaatccaatccagcctga	tgactgttcacattgactgg
	
	Exon 2_1	gttgaggcagcacaaaggtc	gctcctttctcctggggtg
	
	Exon 2_2	caatccctgtcaaggaagtg	ggagtaaccatcaattccatc
	
	Exon 2_3	gagtgtgcttccagcccttg	gagctaactacaccatctgtg
	
	Exon 3	ctctggtaaacaaagcattgtc	cagggagttctaagccaatc
	
*CRB1*	Exon 4	agtaagatgatgccatgggt	tcatttgctataagcgatatgtgt
	
	Exon 5	gacttagcagcttctctgaatt	gtcaagtcatatcccatctcc
	
	Exon 6 1	cctgagctattcatgcacttc	gaagtgagggatgcatgttcc
	
	Exon 6_2	gatattctcctgggctgtacc	gaagagccattggctgaacag
	
	Exon 6 3	ggctcagtttgtaacatagcc	ggagtcgtcgattaaggtaag
	
	Exon 6_4	ccagcgatggagagtggca	ctacaaacgaaggtgtggatg
	
	Exon 6_5	ggtgttgctctgcttaacttc	ctgctctgctctgaggcatg
	
	Exon7_ 1	gtcttccatcccttctgtct	tttgggagagtttggagtca
	
	Exon 7_2	agatttggccaggatgactc	aggccaccaatgtagatgac
	
	Exon 7_3	gactgaacttaatggtggattc	ggtgggtcagtaacatcatc
	
	Exon 8	cagatatgtggtttcaccgtc	gtcgcaacttaactggtgag
	
	Exon 9_1	caatgatcattactattaataacgg	gtgccatcattcactgactgc
	
	Exon 9_2	tcaattgcaaagtggcaaca	ttaactgcaaacagccagtg
	
	Exon 9_3	caatataaagggcctgcaagg	ctgcaactctgtcagagcag
	
	Exon 9_4	cactgtgaactcaacatcgatg	cagtgatgcagagtatagcttc
	
	Exon 10	gaacaagatgaacagctgtgg	gctcagaattctcttccagaag
	
	Exon 11	ccaatgtattcaacagggacc	caactggctcgtcattcatac
	
	Exon 12	cctttgctatagaattcgcatc	gtacagtcatcacattcacag

	Exon 1	ctcaagactgcttccaaacc	tcccaaagccataactcctt
	
	Exon 2	ctatctctgcggactttgag	ggaagccagagaagagagac
	
	Exon 3	cccaaggcagggataagaag	ctaggccctactttgaggag
	
	Exon 4	ttgtcagtaacctctactcctc	atggccattctaagctcca
	
	Exon 5	ggcttgaaaattactggactg	ctgaacatcacctagcactg
	
	Exon 6	cctagggacaaaggtataatg	cacaatacagtaactttctcac
	
*RPE65*	Exon 7	gtatcaaaggtaggcaaagca	cgtttccaaatctgctgcta
	
	Exon 8	gtggcttgagaatcagccct	catcttcttcagaatcacaaac
	
	Exon 9	caagtttgtgattctgaagaag	ccgtaatttccaggaacaatg
	
	Exon 10	cattgcctgtgctcatgtttg	cctgagagagatgaaacattc
	
	Exon 11	gaattctttcctgctcactgag	gagcacatgcttaggaaaactc
	
	Exon 12	caaagatgggttctgatggg	cctcgtcaaggtgagatga
	
	Exon 13	acgaactaacatacagaactgc	tcactttgttccagatagggt
	
	Exon 14	gacattcaatctatagcttggg	gcagacctgaagctgattttc
	
	Exon 2_1	catgggttactcgggcttg	gagaagatggggtcgcaag
	
	Exon 2_2	atcatcccatgggttactcg	gcgatcccggcttctt
	
	Exon 2_3	tcgtgggtccggtgaa	gtagtggatcgtgtcgaagg
	
	Exon 3	ggacggcgccgcgagccaag	tcccctctcccttgccttct
	
	Exon 4	gtgggctgtgaccccgacc	tggtccatggcgattgtctc
	
	Exon 5	ctatcattcccagcctctcc	ttgctgcagacttccatttc
	
	Exon 6	gacggaacttggtgcccttgg	ggaaggaaccaaatttacgga
	
	Exon 7	ctcagcctgacctcaaccca	tcctcctgagagtgcgcctc
	
	Exon 8	gcattctgggacagtgagcc	agaaaccgatggccacctag
	
*GUCY2D*	Exon 9	ccccacattgccctgggcaga	cctgcccccaggacgtcacc
	
	Exon 10	agcaggctgaggctgcctct	cccggtggatcctcgtctgc
	
	Exon 11	ctttctctgagatggctcct	tttagaggaaagagtgaggct
	
	Exon 12	caggccagggtcagaggcagc	ctcaggttgctgacaagcat
	
	Exon 13	cagctttaccagcttccttc	gcaggcagtgaggtcacctg
	
	Exon 14	gaccggctgcttacaca	gctggaggctggtgaag
	
	Exon 15	ggcaatcgcttcgtgtactc	gatgggctggagcctgggaa
	
	Exon 16	ccccgaggccctacctaggt	acctccccgtcttgtccccg
	
	Exon 17	gcatctccacaggtccat	agggtgagctgaggtttg
	
	Exon 18	caaacctcagctcaccctt	tctaagtcagaaagggatcgg
	
	Exon 19	gatgacgtgggccctgccctccc	cttgggtgggacgttctgcag

	Exon 1	gcacacctggaatgttgaa	aaaggtggatgggataggag
	
	Exon 2	gggccttgaacagtgtgtct	tttcccgaaacacagcagc
	
*AIPL1*	Exon 3	agtgagggagcaggattc	tgcccatgatgcccgctgtc
	
	Exon 4	ctcctgcccagggaga	gggagatgtgccacagg
	
	Exon 5	aaagtccaggaaggctatgg	taaggaacctgcagaccaag
	
	Exon 6	ctgggaagggagctgtag	aaaagtgacaccacgatcc
	
	
	Exon 2	gtgcacgtcaccccatggtgagtaac	cagaggtcctccaagagatgaggcc
	
*CRX*	Exon 3	gtagaagggcagggaatgt	ctcctcccatcactctttgt
	
	Exon 4_1	gctggatgcaaagtagacag	ccatgggagaaaggtaggg
	
	Exon 4_2	tctccgagctcctatttcag	gatctaaactgcagggaagc

	Exon 3	ggagaggagcagagaagcag	gcttccagtgcaggtctttg
	
	Exon 4	tcttagtgtgagctcgtgaagg	ttctagtcagagcccccaag
	
	Exon 5	cccagtcccaagctcactta	tagtggggtggatgatggtt
	
*RDH12*	Exon 6	gggcaattatgcaggtctgt	ccctggacattctccacatt
	
	Exon 7	aattggttcacacccagaaga	tgacttcccaagttgctgtg
	
	Exon 8	tcctgagtccctccttctca	tcatcaggcacaaactcagc
	
	Exon 9	gggaccataaagatttccaga	ctttagggttggccttctcc

	Exon 1	tgctgagaaattcctgctacaa	tctgtgaaggccagcaagat
	
	Exon 2	tgagacatctaaagggttcaaaaa	cagtctatcgacatgtttggc
	
	Exon 3	tgtactggggacagaaggcta	aaacgtggctggcacatc
	
	Exon 4	cagcccttcatgttccagtt	ttccctgatcatgctgaaaa
	
	Exon 5	ccaaggttactgattcacttaatttc	cctctgagatggaggaaagg
	
	Exon 6	cgtgatgagaaatgggagaaa	cgagttgtgaggcttggatt
	
*RPGRIP1*	Exon 7	agtgtgctaagtaacagtacct	atttgctccagcaataggc
	
	Exon 8	caaagtcattctttgtgacatctg	ggagcttcgtttttgtcattt
	
	Exon 9	ggaaaatcctcattaatcccaat	attgagtaccaatttccccata
	
	Exon 10	aggtccaggagatgctgaaa	ggatcaagtgaggggattaaa
	
	Exon 11	ttttgttttcggagtgcaag	gttttctaatctcatcatcttccc
	
	Exon 12	agtttctgctgctggcattt	ggacagccattgtgtgtttg
	
	Exon 13	gggtctgcaaggaaatcaaa	atgagaggcacccttcttga
	
	Exon 14	cacaacttggacttccacca	ggggaatacagatggtgtgg
	
	Exon 15	agcaccaatgcagaatttcc	gatgtagctcgctccaaagg
	
	Exon 16	gctcttcctcaccacagatcc	tctgctctgttgctcttgaca
	
	Exon 17	ggtgctgacaaatgctcact	cacatgacactcacagaggga
	
	Exon 18	tcccaaatccctttcttgtg	tgtctgcttctgcttctgct
	
	Exon 19	aaagaaggcaggaaggaagg	cttgaaagcctgatctcgtg
	
	Exon 20	tgaccagacagtggattgga	tgcattttccatcagcttca
	
	Exon 21	tgggttaattggatggcgta	attcaccccacaaaaatcca
	
	Exon 22	ccatgaataccactaatgaaagtct	catcagcacaaaaccaaactc
	
	Exon 23	aaatggaggcaagggaaaag	gggataagatttcaatccactttg
	
	Exon 24	cattcatttagcatccccagt	ggtactggagaaaaatgcctttag

	Exon 1_1	agggcactgtcatgatctcc	ggtaaacatgtcccgctgtg
	
	Exon 1_2	tcagtgccatcagaacagact	ggctggactggcaacaatg
	
	Exon 1_3	cacagcgggacatgtttacc	ggcaggagaattgcttgacc
	
	Exon 1_4	ggaacattgttgccagtcca	cgtggcctttgagttcagtt
	
	Exon 1_5	tctgggcatagcatcacaca	atgctaacaatgtttgagaggca
	
	Exon 2	tttgtccttgctgaccatgc	ggccctagatcaaagcctca
	
*AHI1*	Exon 3	tgggtgacacagcaagactc	tggtcacatacctgaaagctga
	
	Exon 4	actttgggtccttgtcccat	agcaggtccctggtaaatgt
	
	Exon 5	aactgtgcatgaggcaggt	agcaaaccttgagacagcct
	
	Exon 6	agagaaatgaagcataatggcct	acacatcttgcgctattgct
	
	Exon 7	tttgccctttaatgggatgtga	tgacctatcatgtgtcctggt
	
	Exon 8	tggtgcattccagttctttgg	tgccatttgtttgggcaagt
	
	Exon 9	aggtgtggtcatctggttca	cccatcccagtttacatggc
	
	Exon 10	aattgcggacacgaaagaca	gaggagggtcagtggaatgt
	
	Exon 11	tgtgttagcctccattaaacgc	aaactccctgggctcttgg
	
	Exon 12	actgccagatgttccttggt	cagccctaaactgacgttactc
	
	Exon 13	atgccacagtgcaaatggg	acacatgtactgagaggctcat
	
	Exon 14	gcccggccaccatattattc	ggttcattggctgtgttggt
	
	Exon 15	gcaccactggattctaccct	tgtgctgcaaatgtctttggt
	
	Exon 16	gctatcaactagccacattggac	tggcagtgatggctttagagt
	
	Exon 17	ggcctccagaactgtgagaa	ggtgaagaagcagaaacaaagga
	
	Exon 18	tcaactcctgctttaaatcaacctt	gttcagcgtgaaatctggca
	
	Exon 19	ggcatggctgtttgtgtctt	atggaccctccctaactgaatg
	
	Exon 20	cgtctcacttgattccacagc	tcatgttacccaggctggtc
	
	Exon 21	tgaggcagtagaatcgcttga	ggtttgctgttgtctggctt
	
	Exon 22	gagatcgtgccactgcattc	catttacttggcagcagggt
	
	Exon 23	ggcagatgcccttaaatgtc	tcttccactcttttggcaat
	
	Exon 24	agcacaatgaaggaaagcca	tcatcttgtagcaccgaatgtt
	
	Exon 25	cctgtaggacagcactcaaga	acaggctaggcacaccttag
	
	Exon 26	ccttgtccatctgagtcccaa	tcactgtgagtgtgctaccc
	
	Exon 27	ggaatgctaaacgcagcaca	gctgatagcgtagtgaccga
	
	Exon 28	cgtcggtcactacgctatca	tttccctgcgctagctacaa

**Supp. Table S2:** *In-silico* predictions of the novel missense variants and known unclassified variants identified in this study using the Alamut software

				PolyPhen		SIFT						
									
Gene	Nucleotide change	Amino acid change	Domain/Region	Prediction	PSIC score difference	Prediction	Score	Median sequence conservation	Grantham score	Nucleotide conservation	Amino acid conservation	Remarks
*CEP290*	c.5081T>C	p.Leu1694Pro UV	Coiled coil	Possibly damaging	1.995	Affect protein function	0.00*	3.44	98	Weakly conserved (score: 0.0)	Moderately conserved (considering 12 species)	

*CEP290*	c.4696G>C	p.Ala1566Pro UV	Coiled coil	Possibly damaging	1.638	Tolerated	0.10	3.44	27	Highly conserved (score: 1.0)	Highly conserved, up to Frog (considering 12 species)	

*CRB1*	c.929G>A	p.Cys310Tyr	EGF-like 8, extracellular domain	Probably damaging	3.761	Affect protein function	0.00*	3.96	194	Highly conserved (score: 1.0)	Highly conserved, up to Cow (considering 8 species)	Disruption of annotated bond formation site (PolyPhen)

*CRB1*	c.1472A>T	p.Asp491Val UV	Laminin G-like1, extracellular domain	Benign	1.410	Affect protein function	0.01*	3.96	152	Highly conserved (score: 1.0)	Weakly conserved (considering 8 species)	

*RPE65*	c.253C>T	p.Arg85Cys		Probably damaging	2.476	Tolerated	0.05	2.90	180	Highly conserved (score: 1.0)	Moderately conserved (considering 18 species)	

*RPE65*	c.542C>T	p.Pro181Leu		Probably damaging	2.956	Affect protein function	0.04	2.90	98	Highly conserved (score: 1.0)	Highly conserved, up to Fruitfly (considering 18 species)	Located next to metal ion binding site (UniProtKB)

*GUCY2D*	c.587A>T	p.Glu196Val UV	Extracellular domain	Possibly damaging	1.650	Affect protein function	0.00*	4.32	121	Weakly conserved (score: 0.3)	Highly conserved, up to Opossum (considering 10 species)	

*GUCY2D*	c.1724C>T	p.Pro575Leu UV	Cytoplasmic domain	Benign	1.433	Affect protein function	0.00*	4.32	98	Weakly conserved (score: 0.0)	Highly conserved, up to Opossum (considering 10 species)	rs28743021

*GUCY2D*	c.2132C>T	p.Pro711Leu UV	Protein kinase, Cytoplasmic domain	Probably damaging	3.140	Affect protein function	0.00*	4.32	98	Highly conserved (score: 1.0)	Highly conserved, up to Opossum (considering 10 species)	

*GUCY2D*	c.2598G>C	p.Lys866Asn	Cytoplasmic domain	Probably damaging	2.236	Affect protein function	0.00*	4.32	94	Highly conserved (score: 1.0)	Highly conserved, up to Opossum (considering 10 species)	

*AIPL1*	c.341C>T	p.Thr114Ile UV	PPIase FKBP-type	Benign	0.179	Tolerated	0.13	3.34	89	Weakly conserved (score: 0.0)	Moderately conserved (considering 12 species)	rs8069375

*AIPL1*	c.1126C>T	p.Pro376Ser UV		Benign	?	Tolerated	0.48	4.32	74	Weakly conserved (score: 0.0)	Weakly conserved (considering 12 species)	

*CRX*	c.425A>G	p.Tyr142Cys UV		Benign	1.458	Affect protein function	0.00*	4.32	194	Highly conserved (score: 1.0)	Highly conserved, up to Little brown bat (considering 8 species)	rs61748442

*CRX*	c.724G>A	p.Val242Met UV		Benign	1.033	Tolerated	0.20	4.32	21	Weakly conserved (score: 0.2)	Highly conserved, up to Dog (considering 8 species)	RS61748459 VAR_007949

*RDH12*	c.524C>T	p.Ser175Leu		Probably damaging	Prediction basis: sequence annotation	Affect protein function	0.00*	3.61	145	Weakly conserved (score: 0.2)	Highly conserved, up to Fruitfly (considering 15 species)	Disruption of annotated binding site (PolyPhen)

*RDH12*	c.698T>A	p.Val233Asp		Probably damaging	2.616	Affect protein function	0.00*	3.60	152	Weakly conserved (score: 0.0)	Highly conserved, up to Fruitfly (considering 15 species)	

*AHI1*	c.2273A>C	p.His758Pro	WD4	Probably damaging	2.679	Affect protein function	0.03*	3.39	77	Highly conserved (score: 1.0)	Moderately conserved (considering 12 species)	

*AHI1*	c.2433T>G	p.Asn811Lys	WD5	Possibly damaging	1.719	Affect protein function	0.00*	3.39	94	Highly conserved (score: 1.0)	Highly conserved, up to *Tetraodon* (considering 12 species)	

*AHI1*	c.3368C>T	p.Ser1123Phe		Probably damaging	Prediction basis: sequence annotation	Tolerated	0.07	3.67	155	Highly conserved (score: 0.9)	Weakly conserved (considering 12 species)	Disruption of annotated functional site (modified residue) (PolyPhen)

Alamut provides for each variant the HGVS nomenclature and a nucleotide conservation score which was computed at UCSC from 17 vertebrates and has a range between 0 and 1 (http://genome.ucsc.edu/cgi-bin/hgTrackUi?g=multiz17way). For missense variants, Alamut calculates the Grantham distance and automatically fills in queries for PolyPhen and SIFT prediction servers, based on the UniProt protein identifiers and FASTA sequences of several orthologs, respectively. In addition, information on topological as well as functional domains and variations (VAR) was extracted from the UniProtKB database using the following identifiers: O15078 (*CEP290*), P82279 (*CRB1*), Q16518 (*RPE65*), Q02846 (*GUCY2D*), Q9NZN9 (*AIPL1*), O43186 (*CRX*), Q96NR8 (*RDH12*) and Q8N157 (*AHI1*) (http://www.uniprot.org/uniprot/). Variants were designated as “unclassified variant (UV)” if no consensus was seen in all prediction programs used.

* These substitutions may have been predicted to affect function just because the sequences used were not diverse enough.

**Supp. Table S3:** Clinical data of 80 patients with mutation(s) in one of the LCA genes Part I

Gene	Patient n°	Gender	Night Blindness	Photophobia	ERG	Color vision	Fundus aspect	Other features
*CEP290* p.[Cys998X]+[Cys998X]	LCA-1	M			Absent (4mo)		Optic disc pallor Retinal vessel attenuation Normal macula	

*CEP290* p.[Cys998X]+[Cys998X]	LCA-2	M		Mild	Absent (5mo & 1.5yrs)		Pigmentary retinopathy Normal macula (6yrs) Retinal vessel attenuation Salt and pepper alterations Pseudopapilledema HyperAF ring around macula (14yrs)	Eyepoking

*CEP290* p.[Cys998X]+[**Arg108X**]	LCA-3	M	+	+	Absent (4mo)	+ (6yrs)	Normal optic discs Retinal vessel attenuation Normal peripheral retina Beginning of hyperAF around macula (6yrs)	Eyepoking Enophthalmos

*CEP290* p.[Cys998X(+ )**Gln899X**]	LCA-4	F			Absent (2yrs)		Retinal vessel attenuation Tapetal reflex of posterior pole Pseudopapilledema	

*CEP290* p.[Cys998X(+ )Arg1465X]	LCA-5	M			Absent (4mo)			Eyepoking Enophthalmos

*CEP290* p.[Cys998X]+[Lys1575X]	LCA-6	F	-	Strong (since 3.6yrs)	Severe CRD (3.3yrs)	B/Rand G/R	Retinal vessel attenuation White marbleized changes Macular oedema (3.2yrs) Retinal vessel attenuation White marbleized around vascular arcade Discrete RPE alterations No macular reflex (4.9yrs) Extensive peripheral outer retinal atrophy Limited spicular intraretinal pigmentation Relative macular preservation Relatively darker perifoveal ring (18yrs)	Exotropia

*CEP290* p.[Cys998X(+ )Lys1575X]	LCA-7	M	(+) (later in life)	+ (early in life)	Subnormal (1yr) Absent (21yrs)	+ (early in life)	Peripheral salt and pepper intraretinal pigmentation Relative macular preservation (22yrs) Extensive midperipheral and peripheral outer retinal atrophy Limited spicular intraretinal pigmentation Relative macular preservation Development of synchysis scintillans (33yrs) More extensive intraretinal pigmentation (predominantly spicular) Relative macular preservation Hyper AF in central macula and mid- and far periphery Extensive confluent atrophy (49yrs)	Eyepoking Enophthalmos Exotropia Posterior SCP cataract (star shaped) (49yrs)

*CEP290* p.[Cys998X(+ )Lys1575X]	LCA-8	F			Absent (16yrs)	R/G	Optic disc pallor	

*CEP290* p.[Cys998X]+[**Arg1782X**]	LCA-9	M		+	Severe CRD (5mo & 1.2yrs)		Marbleized retinal changes	

*CEP290* p.[Cys998X]+[**Asp128GlufsX17**]	LCA-10	F					Nummular pigmentation Pseudopapilledema Maculopathy	

*CEP290* p.[Cys998X]+[**Glu146GlyfsX17**]	LCA-11	M			Absent (6mo)		Optic disc pallor Yellow confluent peripheral spots	Eyepoking Enophthalmos

*CEP290* p.[Cys998X(+)Arg621IlefsX2]	LCA-12	M			Absent		Retinal vessel attenuation Peripheral pepper and salt alterations Macular RPE alterations (6mo)	Enophthalmos

*CEP290*	LCA-13	F			Absent		Optic disc pallor	

p.[Cys998X(+)**Leu1141PhefsX5**]					(4mo)		Retinal vessel attenuation	

*CEP290* p.[Cys998X]+[**Thr1334IlefsX2**]	LCA-14	F			Absent (5mo)		Granular pigment alterations Normal macula (7mo)	Eyepoking Enophthalmos Cataract

*CEP290* p.[Cys998X(+)Glu1656AsnfsX3]	LCA-15 (Perrault, Delphin et al. 2007)	M		-	Absent		Retinal vessel attenuation (7mo) Hyperaemic optic disc Marbleized, white spots Nummular pigmentation (2yrs)	Eyepoking Enophthalmos

*CEP290* p.[Cys998X]+[Ala1832ProfsX19]	LCA-16	M			Absent (6mo & 10mo)		Yellow spots	Eyepoking

*CEP290* p.[Cys998X]+[**Lys1840ArgfsX5**]	LCA-17	M			Absent (5mo)		Full optic disc with no apparent excavation Marbleized fundus changes in the midperiphery Normal macula (8yrs)	Eyepoking

*CEP290* p.[Cys998X]+[**Glu1956GlyfsX9**]	LCA-18	M		+ (early in life)	Absent (3mo)		Full optic discs Limited outer retinal atrophy Midperipheral salt and pepper alterations Normal macula (15yrs)	Eyepoking Enophthalmos Keratoconus with acute hy drops (OD>OS)(13yrs)

*CEP290* p.[Cys998X]+[**Splice defect**]	LCA-19	M	+	+	Absent (3mo)		Very small optic disc excavation Retinal vessel attenuation Mild midperipheral salt and pepper alterations Bull's eye maculopathy with preservation of central macula, surrounded by concentric area of more pronounced outer retinal atrophy (14yrs)	Eyepoking Enophthalmos

*CEP290* p.[Cys998X(+)**Splice defect**]	LCA-20 (Yzer, Leroy et al. 2006)	F	-	-	Absent (9yrs)		Optic disc pallor Retinal vessel attenuation RPE alterations maculopathy unknown (3mo)	Eyepoking Enophthalmos Cataract with lens luxation Keratoconus (OD) Enucleation because of phacolytic glaucoma (OD)

*CEP290* p.[Cys998X]+[**Splice defect**]	LCA-21	M	+		Absent		Optic disc pallor Retinal vessel attenuation	

*CEP290* p.[Cys998X(+)**Splice defect**]	LCA-22	M	-	-	Absent (4mo & 9mo)		Normal optic discs White retinal spots Tapetal reflex	

*CEP290* p.[Cys998X(+)**Splice defect**]	LCA-23	F			Absent (5mo& 1.5yrs)		Salt and pepper alterations Normal macula (5mo) Pinkish optic disc Mild retinal vessel attenuation RPE alterations (yellowish dots) (6yrs)	Eyepoking Enophthalmos

*CEP290* p.[Lys1575X(+)Lys1575X]	LCA-24	M						

*CEP290* p.[Lys1575X]+[**Ala1566Pro**]	LCA-25	M	-	Strong	Severe CRD (1.1yrs)		No RPE alterations (4mo) Retinal vessel attenuation Discrete RPE alterations (1.10yrs) Retinal vessel attenuation HyperAF ring around macula (8yrs) Retinal vessel attenuation Salt and pepper alterations Mild macular pigment epithelial alterations (10yrs)	Eyepoking Enophthalmos Exotropia

*CEP290* p.[**Leu1694Pr o**]+[=, **splice site**]	LCA-26		-		Absent (4.5yrs)		No retinal vessel attenuation No hyperpigmentation Midperipheral reticular aspect, especially around vascular arcades Assymetric ectopia foveae OS>OD (3yrs) Normal optic disc Deep intraretinal white spots along vascular arcades HyperAF ring around macula No pigmentation/atrophy (5.4yrs)	Strabismus

*CEP290* p.Cys998X	LCA-27	M			Subnormal (5mo & 1yr & 7yrs)	+	Optic disc pallor No RPE alterations Normal macula	

*CEP290* p.[Trp7Cys]+[ Trp7Cys]	SLS-1	F			Absent (2mo)		Abnormal RPE	

*CEP290* p.[Lys1575X( +)Arg1465X]	SLS-2	F	+			+ (12yrs)		Keratoconus Cataract

*CEP290* p.[Lys1575X( +)Arg1465X]	SLS-3	M						Eyepoking

*CEP290* p.[Lys1575X( +)Arg1465X]	CORS-1 (Brancati, Barrano et al. 2007)	F						Enophthalmos

*CEP290* p.[Gln1265X] +[Splice defect]	LCA-JS-1	M		-	Absent (first year of life)		Optic disc pallor Salt and pepper alterations Mild spicular intraretinal pigmentation	

*CEP290* p.[**Thr2457Al afsX27]+[Thr 2457AlafsX27**]	LCA-JS-2II-1	F	+	+	Absent		Normal optic discs Retinal vessel attenuation Salt and pepper alterations	



	LCA-JS-2II-1	M		+	Absent			

*CEP290* p.[Leu2448Th rfsX8(+)**Splice defect]**	LCA-JS-3	M		+	Absent (4mo & 1.4yrs)		Optic disc pallor Retinal vessel attenuation	

*CRB1* p.[Lys801X(+)Lys801X]	LCA-28	M					Marbleized fundus changes	

*CRB1* p.[Lys801X(+)Gln362X]	LCA-29 (Yzer, Leroy et al. 2006)	M	+	Mild	Absent (1yr&30.6yrs)	Basic Severe R-G andB-Y deficienty	Optic disc pallor Retinal vessel attenuation Perivascular fibrosis No clear PPRPE Fine intraretinal white flecks Nummular intraretinal pigmentation Macular atrophy (30.6yrs)	Eyepoking (mild) Enophthalmos (mild) Posterior subcapsular cataract (OS>OD)

*CRB1* p.[Lys801X]+[Arg764Cys]	LCA-30	M						

*CRB1* p.[Lys801X]+[Cys896X]	LCA-31 (Yzer, Leroy et al. 2006)	M	+	-	Absent (3mo)	Basic	Retinal vessel attenuation Outer retinal atrophy RPE defects around vascular arcade Perimacular atrophic spots (3mo) Normal optic discs Perivascular fibrosis No clear PPRPE Outer retinal atrophy Small white dots Nummular hyperpigmentation Pseudopapilledema Macular atrophy (8yrs)	Eyepoking Enophthalmos Exotropia

*CRB1* p.[Lys801X(+)Cys896X]	LCA-32 (Yzer, Leroy et al. 2006)	F	+	(+ after keratoconus)			Depigmentation around macula (15yrs) Macular aplasia: vessels of choroid become apparent (17yrs) Normal optic disc Retinal vessel attenuation Perivascular fibrosis No clear PPRPE Outer retinal atrophy White retinal spots Peripheral intraretinal pigment migration, predominant nummular Some midperipheral lipofuscin depositions Macular aplasia Pseudopapilledema (33yrs)	Eyepoking Enophthalmos Esotropia Keratoconus with acute hydrops and rupture of Descemet membrane (20yrs)

*CRB1* p.[Lys801X(+)Cys948Tyr]	LCA-33	F	+		Absent (9mo&1.7yrs)		Pale and slightly swollen optic disc Diffuse outer retinal atrophy Small whitish deep intraretinal flecks Nummular intraretinal pigmentation Limited macular atrophy	

*CRB1* p.[Lys801X]+[**Splice defect**]	LCA-34	F		+	Absent (4mo & 1.5yrs)		Retinal vessel attenuation Macular pigmentation Pseudopapilledema	Eyepoking Strabismus

*CRB1* p.[Cys948Tyr( +)Cys948Tyr]	LCA-35	F						Keratoconus (OS)

*CRB1* p.[Cys948Tyr( +)Glu1330X]	LCA-36	F					Optic disc pallor Retinal vessel attenuation Nummular intraretinal pigmentation Macular alterations Coats reaction	Enophthalmos Cataract Glaucoma (neovascular with OD seclusio pupillae and anterior synechiae)

*CRB1* p.[Cys948Tyr] +[Splice defect]	LCA-37	M	+					

*CRB1* p.[Cys948Tyr] +[Splice defect]	LCA-38	M	+	-	Absent (9.1yrs)	Basic (until 9yrs) Disturbed (after 9yrs)	Optic disc pallor with irregular shape Retinal vessel attenuation Peripheral salt and pepper alterations (6yrs) Retinal vessel attenuation with tortuous aspect Extensive peripheral and macular outer retinal atrophy Small white deep intraretinal flecks Nummular intraretinal pigment migrations Yellowish hue of the macula 6 astrocytoma-like retinal excrescences superior to right macula Pseudopapilledema with prominent sheathing of blood vessels near optic discs (18yrs)	Eyepoking Enophthalmos Exotropia

*CRB1* p.[Cys948Tyr] +[Splice defect] (LCA-39a) and p. [Splice defect]+[Splic e defect] (LCA-39b)	LCA-39a	F	+	+	Absent (4mo)	Basic (early in life) Declining at the age of 12yrs Absent (15.1 1yrs)	Optic disc pallor Retinal vessel attenuation Salt and pepper aspect Macular aplasia Total chorioretina atrophy in the central macula Pseudopapilledema Atrophic macular region with pigment near border (3yrs) Total atrophy of retina and choriocapillaris Midperipheral small white dots Midperipheral small nummular pigmentation	Eyepoking Enophthalmos Esotropia
	
	LCA-39b	M		+	Absent (8yrs& 16yrs)	-	Optic disc pallor Small excavation optic disc (OD, not OS) Retinal vessel attenuation Extensive peripheral outer retinal atrophy Limited nummular intraretinal pigmentation Macular yellowish atrophy (OD>OS) (16yrs)	

*CRB1* p. [Splice defect(+)Splic e defect]	LCA-40	M	+	-	Absent (2yrs)		No optic disc excavation No perivascular sheathing Severe outer retinal and central macular atrophy Small deep intraretinal white flecks Extensive nummular intraretinal pigmentation (11yrs)	Cataract (OS>OD) Retinal detachment (OD: partial -inferior, OS: total)

*CRB1* p.[**Leu814Arg fsX23**]+[**Cys1 240ProfsX24**]	LCA-41 II-1	F	+	Mild	Absent (31yrs)	Disturbed	Hyperaemic optic disc Retinal vessel attenuation Small pigment clumps scattered through retina Pseudopapilledema Normal macula (12yrs) Macular atrophy with pigment clumping (21yrs) Macular pseudocoloboma (23yrs) Limited perivascular sheathing around optic disc Extensive outer retinal atrophy Small white intraretinal flecks Pronounced nummular intraretinal pigmentation Central macular atrophy (36yrs)	Eyepoking Enophthalmos Esotropia
	
	LCA-41 II-2	F	+	-	Absent (24yrs)	Disturbed	Beginning macular atrophy with pigment alterations (10yrs) Extensive peripheral outer retinal atrophy Extensive macular atrophy Extreme hyperpigmentation around central macula Pronounced peripapillary perivascular fibrosis Relative sparing of small retinal area just nasal to the optic disc (used for fixation) Pronounced mid and far-peripheral nummular intraretinal pigmentation (24yrs)	Eyepoking Enophthalmos Esotropia SCP Cataract (complete, OS)

*CRB1* p.[Trp1293X( +)Trp1293X]	LCA-42	F		+	Absent (2yrs)		White retinal spots Salt and pepper pigmentation Maculopathy (2yrs)	Keratoconus Cataract (OD)

*CRB1* p.[Cys948Tyr] +[Lys801X]	EORD-1 II-1	F	+	-	CRD		Optic disc pallor Retinal vessel attenuation Salt and pepper alterations Nummular intraretinal pigmentation (over 360°) No maculopathy	

	EORD-1 II-2	M	+	Mild	CRD		Optic disc pallor Salt and pepper alterations No maculopathy	

*CRB1* p.[Cys948Tyr(+)**Cys310Tyr**]	EORD-2	M					Optic disc pallor Peripheral nummular intraretinal pigmentation No maculopathy	Cataract

*CRB1* p.[Cys948Tyr]+[**Asp491Val**]	EORD-3	F	+ (since the age of 5)	Strong (since early age)	Absent (4.10yrs)		Hyperaemic optic disc Retina vessel attenuation Midperipheral pigment alterations Pseudopapilledema Bull's maculopathy (4yrs)	

*CRB1* p.[Gln362X (+)Gln362X]	EORD-4	M			Absent (5yrs)		Peripheral nummular intraretinal pigmentation No maculopathy	Cataract

*CRB1* p.[Arg764Cys (+)Arg764Cys ]	EORD-5	F	+ (since the age of 13-14)	-	Absent (12yrs)	Normal (12yrs)	Pseudopapilledema Maculopathy (edema) Coats reaction (OD) Mid-peripheral spicular intraretinal pigmentation Excavation optic disc (OS)	SCP Cataract (OD) Glaucoma

*RPE65* p.[Arg44Gln] +[Arg44Gln]	LCA-43	F	Strong (since the age of 13-14)	(searches for light)	Absent (7mo & 2yrs)	R/B(not R/Y) (4yrs)	Discrete retinal vessel attenuation Normal fundus Very small white intraretinal flecks in mid- and far periphery No preretinal fibrosis (4yrs)	Esotropia

*RPE65* p.[**Pro181Leu**]+[**Pro181Leu**]	LCA-44	M	+	(searches for light)	Absent (5mo & 4yrs)	Disturbed	Retinal vessel attenuation Discrete RPE alterations (5mo) Optic disc pallor Retinal vessel attenuation Mild thinning of inferior retina Limited midperipheral intraretinal pigmentation Well-preserved macula (7yrs)	

*RPE65* p.[Arg234X]+[**Trp331dup**] (LCA-45a) and p.[**Trp331dup**]+[**Trp331dup**] (LCA-45b)	LCA-45a LCA-45b	F					Optic disc pallor Depigmentations and round hyperpigmentations	

*RPE65* p.Leu341Ser **p.Ser121LeufsX6 (*de novo*)**	LCA-46	F					Retinal vessel attenuation Limited but clear peripheral outer retinal atrophy No intraretinal pigmentation Relative preservation of essentially normal macula (21yrs)	

*RPE65* p.[Phe530Leu fsX40]+[Phe5 30LeufsX40]	LCA-47	M	+	(searches for light)	Absent (9mo & 9yrs)	G/B R/0/P	Optic disc pallor Retinal vessel attenuation No pigmentation (5yrs) Normal optic discs Retinal vessel attenuation Discrete retinal thinning Cellophane maculopathy Limited macular pigment alterations Peripheral hypopigmentation Small discrete peripheral white flecks Total absence of AF (9yrs)	Eyepoking Cataract (very limited posterior lens opacification) Semimydriasis (ODS)

*RPE65* p.[Phe530Leu fsX40]+[Arg1 24X]	LCA-48	M			Absent (4mo & 1yr)	Basic	Optic disc pallor Retinal vessel attenuation Outer retinal atrophy especially in inferior midperiphery Relative sparing of the macula. Mild preretinal macular fibrosis	

*RPE65* p.[Phe530Leu fsX40]+[Splic e defect]	LCA-49	M	Strong	(searches for light)	Absent (1.9yrs)	Basic	Retinal vessel attenuation No pigment alterations Normal macula (1yr&4.3yrs) Peripheral pigment alterations (6yrs) Normal optic disc Limited retinal vessel attenuation Peripheral RPE alterations without intraretinal pigment Very limited foveal pigment alterations	Semimydriasis

*RPE65* p.[**Arg296LysfsX7**]+[Splice defect]	LCA-50	F			Absent (5mo)		Normal vessels Mild RPE alterations No hyperpigmentation Normal macula	Esotropia Hypertropia

*GUCY2D* p.[Pro130Leuf sX36 (+)Splice defect]	LCA-51	M			Absent (11mo&3yrs)		Normal fundus	Eyepoking

*GUCY2D* p.[Phe565Ser(+)Phe565Ser]	LCA-52	M			Absent (6mo)		Normal fundus	Eyepoking Enophthalmos

*GUCY2D* p.[Arg768Trp(+)Arg768Trp]	LCA-53	M						

*GUCY2D* p.[Arg768Trp] +[Phe565Ser]	LCA-54	F	+ (since early age)	+ (since the age of2.6)	Absent (3mo)		Normal fundus (1yr & 3.2yrs)	Eyepoking Enophthalmos Esotropia

*GUCY2D* p.[Arg768Trp] +[Lys866Asn]	LCA-55	M			Absent (4.4yrs)	-	Pseudopapilledema Essentially normal fundus Limited peripheral salt and pepper alterations Limited hyperAF of the central macula (13yrs)	Eyepoking

*GUCY2D* p.[**Glu196Val**]+[**Pro711Leu**]	LCA-56	F	-	Strong	Absent (3mo)	Basic Strong R/G andB/Y defect	Optic disc pallor Retinal vessel attenuation Limited peripheral outer retinal atrophy No intraretinal pigmentation Normal macula Foveolar yellowish atrophy No hyper- or hypoAF (25yrs)	Eyepoking Enophthalmos Esotropia Keratoconus with acute hydrops (OD>OS) (16yrs)

*GUCY2D* p.Pro575Leu	LCA-57	F	+		Absent (3.3yrs)	Severely disturbed	Optic disc hypoplasia Bull's maculopathy	

*AIPL1* p.[Trp278X]+[ Trp278X]	LCA-58 (Yzer, Leroy et al. 2006)	F		Mild	Absent (3mo & 1yr)		Bull's eye maculopathy, diffuse RPE alterations; limited intraretinal pigment migration of spicular type; sub- or deep intraretinal fine white deposits predominantly along vascular arcades	

*AIPL1* p.[Trp278X (+)Trp278X]	LCA-59	M		+ (since the age of4.10)	Absent (2yrs)		Retinal vessel attenuation No intraretinal pigmentation Macular pigment alterations (5yrs)	Eyepoking

*AIPL1* p.[Trp278X (+)Trp278X]	LCA-60	M					Normal optic discs Retinal vessel attenuation Peripheral outer retinal atrophy Spicular intraretinal pigmentation Total outer retinal aplasia of central macula, surrounded by thin rim of hyperplastic RPE (28yrs)	

*AIPL1* p.[Trp278X]+[ Trp278X]	LCA-61	F	+	Strong (after first decade)	Absent (3mo & 6mo)		Limited optic disc pallor Retinal vessel attenuation Extensive outer retinal atrophy Mid and far-peripheral spicular pigmentation Better preserved macula with central yellow atrophy (19yrs)	Eyepoking Esotropia

*AIPL1* p.[Thr114Ile; Pro376Ser]	LCA-62	F			Absent (1.4yrs)		Optic disc pallor Retinal vessel attenuation	Eyepoking

*CRX* p.Tyr142Cys	LCA-63	M			Absent (6mo)			Eyepoking

*CRX* p.Val242Met	LCA-64	M						

*RDH12* p.[Ala269Glyf sX2]+[**Val233Asp**]	EORD-6	M					Optic disc pallor Retinal vessel attenuation Salt and pepper alterations	

*RDH12* p.[Ala269Glyf sX2]+[**Ser175Leu**]	EORD-7	M	+ (since early age)	+	Absent (3.5yrs)	Basic (5.10yrs)	Limited retinal vessel attenuation Better preservation of the chorioretina in the posterior pole than in the periphery Clear retinal pigment epithelium alterations Peripheral areas of preserved chorioretina alternating with areas of total atrophy with predominant spicular intraretinal pigmentation (5yrs) Retinal vessel attenuation Yellowish discoloration of central macula More prominent spicular intraretinal pigmentation Areas with complete preservation of peripheral chorioretina (19yrs)	Esotropia

*RDH12* p.[Ala269Glyf sX2(+)Ala269 GlyfsX2]	EORD-8							

*RPGRIP1* p.Arg890X	LCA-65			Strong				

**Supp. Table S3:** Clinical data of 80 patients with mutation(s) in one of the LCA genes Part II

				BCVA (age)		Refraction (age)						
										
Gene	Patient n°	Gender	Nyst	OD	OS	OD	OS	VF	MTS	Neurological Features	Kidney	Other Features
*CEP290* p.[Cys99 8X]+[Cys 998X]	LCA-1	M		LP?	LP?	+5	+5		Normal MRI			

*CEP290* p.[Cys99 8X]+[Cys 998X]	LCA-2	M	+	20/600 (1yr&5yrs)	20/600 (1yr&5yrs)	+7	+7			-		

*CEP290* p.[Cys99 8X]+[Arg 108X]	LCA-3	M	+	2/24 (6yrs)	2/24 (6yrs)	+7 (3mo) +8.25 (6yrs)	+7 (3mo) +8.25 (6yrs)		MRI: Broadened supertentorial ventricular system without signs of intracranial hypertension (11mo)	Mild MR Autism	RDI Daytime incontine nce Normal kidney US (6.10yrs)	Growth retardation (length and weigth) Prematurity (36w)

*CEP290* p.[Cys99 8X(+)**Gln 899X**]	LCA-4	F	+	NLP (since birth)	NLP (since birth)					-		

*CEP290* p.[Cys99 8X(+)Arg 1465X]	LCA-5	M		NLP (1.8yrs&8yrs)	NLP (1.8yrs& 8yrs)	+6 (4mo)	+6 (4mo)		TDM brains: modest cortical atrophy with limited subdural bifrontal fluid collection (4mo) EEG: normal (4mo)			MEI

*CEP290* p.[Cys99 8X]+[Lys 1575X]	LCA-6	F	+	1.5/24 (3.6yrs) 1/20 (18.5yrs)	3/36 (3.6yrs) 2/10 (18.5yrs)	+4 (18yrs)	+4 (18yrs)	Reduced	MRI: slightly broadened lateral ventricles (3.4yrs)	Learning disability	Normal kidney US, normal kidney function (3yrs)	

*CEP290* p.[Cys99 8X(+)Lys 1575X]	LCA-7	M	+	CF at 2m (21yrs) LP with incomplete loc (49yrs)	CF at 2m (21yrs) HM at 2m (49yrs)	+3.5 (42yrs)	+3.5 (42yrs)	30° (35yrs & 49yrs)		-		

*CEP290* p.[Cys99 8X(+)Lys 1575X]	LCA-8	F	+	1/60 (6yrs) HM 2m (30yrs)	1/36 (6yrs) HM 1.5m (30yrs)	+5	+5	5°		-		

*CEP290* p.[Cys99 8X]+[**Arg 1782X**]	LCA-9	M	+	1/20 (6yrs)	1/20 (6yrs)	+2.25	+2.25	10° (6yrs)	Normal MRI	-		

*CEP290* p.[Cys99 8X]+[**Asp 128GlufsX17**]	LCA-10	F	+	NLP	NLP	+4	+4		Normal MRI (10yrs)	-	Normal kidney US (10yrs)	Obesity

*CEP290* p.[Cys99 8X]+[**Glu 146GlyfsX17**]	LCA-11	M	REM	NLP	NLP	+8	+8		Normal MRI	-		

*CEP290* p.[Cys99 8X(+)Arg 621IlefsX2]	LCA-12	M	+	NLP	NLP	+10 (6mo)	+10 (6mo)			-	Normal kidney US (1yr)	

*CEP290* p.[Cys99 8X(+)**Leu 1141PhefsX5**]	LCA-13	F		NLP	NLP	+8	+8		Normal CT scan MRI: 2 atypical white matter lesions (17yrs-19yrs)	MR Epilepsy		

*CEP290* p.[Cys99 8X]+[**Thr 1334IlefsX2**]	LCA-14	F	+	LP? (7mo) NLP (7.8yrs)	LP? (7mo) NLP (7.8yrs)				MRI: slightly broadened lateral ventricles (5mo)	-		Limited ventricle septum defect (VSD): slow closure, Asthma Familial palatoschisis Brother died from SIDS

*CEP290* p.[Cys99 8X(+)Glu 1656Asnf sX3]	LCA-15 (Perrault, Delphin et al. 2007)	M	+	LP	LP	Hypermetr ia hypermetr opic (strong)	Hypermetr ia hypermetr opic (strong)		Normal MRI (1.3yrs)	Dev del Autism?		Discrete scoliosis

*CEP290* p.[Cys99 8X]+[Ala 1832ProfsX19]	LCA-16	M	+	NLP	NLP	+8	+8			-		Obesitas

*CEP290* p.[Cys99 8X]+[**Lys 1840ArgfsX5**]	LCA-17	M	+	LP No loc	LP No loc	+7	+7			Autism Normal IQ		Carrier of a non-pathogenic translocation : 45,XY,t(13;14) (father has the same)

*CEP290* p.[Cys99 8X]+[**Glu 1956GlyfsX9**]	LCA-18	M	+	LP No loc (early in life)	LP No loc (early in life)			Conc constr	Normal CT scan (5mo)	Non-verbal learning disability Ataxia (Mild) Dyspraxia (10yrs) Balance and coordinatio n problems	Normal kidney US (5mo)	

*CEP290* p.[Cys99 8X]+[**Splice defect**]	LCA-19	M	+	NLP (since birth)	NLP (since birth)				MRI: Frontal and temporal cortical atrophy	Autism Mild-moderate MR Verbal IQ = 55 Coordinati on problems	Normal kidney US (6mo)	

*CEP290* p.[Cys99 8X(+)**Splice defect**]	LCA-20 (Yzer, Leroy et al. 2006)	F	+	<1/20 (45yrs) NLP (54yrs)	<1/20 (45yrs) NLP (54yrs)					Severe MR		Meningitis (6w)

*CEP290* p.[Cys99 8X]+[**Splice defect**]	LCA-21	M	+	LP	LP	+5	+5		Normal CT scan	Mild MR ADHD Movement abnormalities		Chrom dupl 14q24-32.3 (< mother: carrier of a balanced translocation)

*CEP290* p.[Cys99 8X(+)**Splice defect**]	LCA-22	M	-	LP	LP					-		

*CEP290* p.[Cys99 8X(+)**Splice defect**]	LCA-23	F	+	LP?	LP?				(1.2yrs)	Severe MR Dev Del Epilepsy Axial hypotonia (mild)	Kidney US: hyperden sity (3yrs) No other signs of NPHP (17yrs)	Hyperlax ligaments Hyperlordosis

*CEP290* p.[Lys157 5X(+)Lys 1575X]	LCA-24	M								Severe MR		

*CEP290* p.[Lys157 5X]+[**Ala 1566Pro**]	LCA-25	M	+	20/600 (9mo) 1/60 (5yrs)	20/600 (9mo) 1/24 (5yrs)	+3.5 (9yrs)	+3.5 (9yrs)	10°- 15° paracent ral	Normal MRI (4mo)	-		

*CEP290* p.[**Leu1694Pro**]+[=,**splice site**]	LCA-26		+	5/60 (3yrs) 1/10 (4.5yrs)	5/60 (3yrs) 1/10 (4.5yrs)	+3.25 (4.5yrs)	+3 (4.5yrs)					

*CEP290* p.Cys998X	LCA-27	M	+	0.16	0.16			50°		-		

*CEP290* p.[Trp7C ys]+[Trp7Cys]	SLS-1	F	+	No reaction on light (2mo)	No reaction on light (2mo)	>+4	>+4		-?	-No ataxia/hypotonia	UTI RDI CKD5 (5yrs) RTx (6yrs)	Glue ear Clinodactyly Sibling died shortly after birth (enlarged kidneys, chrom 6 defect)

*CEP290* p.[Lys157 5X(+)Arg 1465X]	SLS-2	F	+	LP with loc	LP with loc			<20° (14yrs)	Broadened 4th ventricle	Mild MR Balance problems	Diagnosi s renal insufficie ncy (30yrs) RDI Peritonea l dialysis (34yrs) Kidney transplan t (34yrs)	Syncopes Scoliosis

*CEP290* p.[Lys157 5X(+)Arg 1465X]	SLS-3	M		NLP	NLP					Moderate MR Severe autism Mild ataxia	RDI (7yrs) Kidney US: hyperden sity (13yrs) CKD5 (16yrs) Peritoneal dialysis (since the age of 17)	Recurrent OM

*CEP290* p.[Lys157 5X(+)Arg 1465X]	CORS-1 (Brancati, Barrano et al. 2007)	F	+	LP with loc	LP with loc	+10, +12	+10, +12		+	Severe MR Ataxia Balance problems	RDI (6yrs) Kidney US: hyperden sity (6yrs) CKD5 (14yrs) Deceased (16yrs)	Scoliosis Recurrent OM Congenital chylothorax Alternating tachypnea Syncopes

*CEP290* p.[Gln126 5X]+[Splice defect]	LCA-JS-1	M	+	1/100	1/100	+5	+5	tubular	+ (4yrs)	Hypotonia Walking problems		

*CEP290* p.[**Thr24 57AlafsX27**]+[**Thr2457AlafsX27**]	LCA-JS-2 II-1	F	+	1.5/10	1/10	+5	+5	10°	+	Walking problems		

	LCA-JS-2 II-2	A	+	1/20	1/20	+4.5	+3.5	10°	+	Mild MR Walking problems		

*CEP290* p.[Leu24 48ThrfsX 8(+)**Splice defect**]	LCA-JS-3	M	REM	NLP (since birth)	NLP (since birth)				+	Severe psychomot or retardation Hypotonia No ataxia	Normal kidney US (1yr)	Scoliosis

*CRB1* p.[Lys801X(+)Lys801X]	LCA-28	M		1/100	1/100							

*CRB1* p.[Lys801X(+)Gln362X]	LCA-29 (Yzer, Leroy et al. 2006)	M	+	20/600 (1yr) 1/30 (7.9yrs) 1/40 (25 yrs)	20/600 (1yr) <1/50 (7.9yrs) 1/40 (25 yrs)	+2.25 (30.6yrs) (astigm)	+1.88 (30.6yrs) (astigm)	OD:central residual visual field, OS: temporal inferior and partially superior visual field intact (30.6yrs)		-		

*CRB1* p.[Lys801X]+[Arg7 64Cys]	LCA-30	M										

*CRB1* p.[Lys801X]+[Cys8 96X]	LCA-31 (Yzer, Leroy et al. 2006)	M	+	HM on 20 cm (3mo & 6yrs)	HM on 20 cm (3mo & 6yrs)	+10	+9.5			-		

*CRB1* p.[Lys801X(+)Cys8 96X]	LCA-32 (Yzer, Leroy et al. 2006)	F	+	1/100 (1yr) < 1/600 (35yrs)	1/100 (1yr) < 1/600 (35yrs)	+4 (19yrs)	+4 (19yrs)	40°; remaining temporal crescent (19yrs)				Asthma Torticollis (to the left)

*CRB1* p.[Lys801X(+)Cys9 48Tyr]	LCA-33	F	+	CF at 1m (until 8yrs) HM at 0.5m (9yrs)	CF at 1m (until 8yrs) CF at 10cm (9yrs)	+4	+4	10°		-		

*CRB1* p.[Lys801X]+[**Splice defect**]	LCA-34	F	+	20/800 (1.11yrs)	20/800 (1.11yrs)	+5	+5	Remaining island in peripher al field		-		

*CRB1* p.[Cys94 8Tyr(+)Cys948Tyr]	LCA-35	F		LP	LP							

*CRB1* p.[Cys94 8Tyr(+)Glu1330X]	LCA-36	F	+	LP	LP					-		

*CRB1* p.[Cys94 8Tyr]+[Splice defect]	LCA-37	M	+	LP (decreased since the age of 13)	LP (decreased since the age of 13)				Normal CT scan	-		

*CRB1* p.[Cys94 8Tyr]+[S plice defect]	LCA-38	M	+	0.07 LP with localisation (17yrs)	0.07 LP with localisation (17yrs)	+9	+9	10°	Normal CT scan (5.9yrs)	-		

*CRB1* p.[Cys94 8Tyr]+[Splice defect] (LCA-39a) and p. [Splice defect]+[ Splice defect] (LCA-39b)	LCA-39a	F	+	1.5/36 (4.9yrs) HM1m (15.1 1yrs)	2/36 (4.9yrs) HM1m (15.1 1yrs)	+4.75	+5.25	10°		-		
	
	LCA-39b	M		0.08	0.08			10°-20°		-		

*CRB1* p. [Splice defect(+) Splice defect]	LCA-40	M	+	0.08 (3yrs) LP (22 yrs)	0.08 (3yrs) NLP (22 yrs)			<10°	Normal CT scan	-		

*CRB1* p.[**Leu81 4ArgfsX23**]+[**Cys1240ProfsX24**]	LCA-41 II-1	F	+	3/10 (8yrs) CF at 15cm (37yrs)	3/10 (8yrs) CF at 15cm (37yrs)	+9	+9	30-50°		-		
	
	LCA-41 II-2	F	+	1/20 (14yrs) 1/20 (28yrs)	1/100 (14yrs) CF at 50cm (28yrs)	+4.5 Astigm	+1.5 Astigm	50-60° horizont ally 70° vertically		-		

*CRB1* p.[Trp129 3X(+)Trp 1293X]	LCA-42	F		2/10 (2yrs) LP	4/10 (2yrs) LP	+5	+5	10°		-		

*CRB1* p.[Cys948Tyr]+[Lys801X]	EORD-1 II-1	F	-	1/50	1/50	+6	+6	60°		-		
	
	EORD-1 II-2	M		1/50	1/20	+3	+3	10°		-		

*CRB1* p.[Cys94 8Tyr(+)**C ys310Tyr**]	EORD-2	M		LP	NLP	+	+	10°		-		

*CRB1* p.[Cys94 8Tyr]+[**A sp491Valt**]	EORD-3	F	+	2/10 (4yrs) 1/20 (11.9yrs)	2/10 (4yrs) 1/20 (11.9yrs)	+4.5 (10.10yrs)	+4.5 (10.10yrs)	Complet e (4yrs) 15°-30° (11yrs)	MRI: subcortical white matter lesions, frontal (right) in centrum semi-ovale	Learning disability (but normal IQ)		

*CRB1* p.[Gln362X (+)Gln36 2X]	EORD-4	M	+	3/10 (16yrs) LP	3/10 (16yrs) LP	+6	+6	10°		-		

*CRB1* p.[Arg764Cys(+)Arg764Cys]	EORD-5	F	-	5/10 (until 5yrs) LP no loc (18.4yrs)	7/10 (until 5yrs) HM (18.4yrs)	+2D (12yrs) +5D	+3D (12yrs) +5D	30° (12yrs) Absent (19yrs)		-	Normal kidney US	

*RPE65* p.[Arg44 Gln]+[Ar g44Gln]	LCA-43	F	+	1/20	1/20	+4.5 (7mo)	+4.5 (7mo)	Conc constr	Normal MRI	MR Autism		Obesitas

*RPE65* p.[**Pro181Leu**]+[**Pro181Leu**]	LCA-44	M	+	1/10 (4yrs & 9yrs)	3/10 (4yrs & 9yrs)	-6	-6		Normal CT scan andEEG (5mo)	Behavioural anomalies	Normal kidney US (2yrs)	Mild perceptive hearing loss (Cx26 negative)

*RPE65* p.[Arg23 4X]+[**Trp 331dup**] (LCA-45a) and p.[**Trp331dup**]+[**Trp331dup**] (LCA-45b)	LCA-45a	F		<1/20	<1/20							
	
	LCA-45b											

*RPE65* p.Leu341Ser **p.Ser121 LeufsX6 (*de novo*)**	LCA-46	F										

*RPE65* p.[Phe530LeufsX40]+[Phe530LeufsX40]	LCA-47	M	+	1/10 (5yrs) 5/100 (9yrs)	1/10 (5yrs) 5/100 (9yrs)	+4 (9yrs)	+4.5 (9yrs)	Moderate conc constr		Attention deficit disorder		

*RPE65* p.[Phe530LeufsX40]+[Arg124X]	LCA-48	M	+	15/100 (5yrs) 2/10 (10yrs)	12/100 (5yrs) 2/10 (10yrs)	-2.25	-2.5	40°		-		

*RPE65* p.[Phe530LeufsX40]+[Splice defect]	LCA-49	M	+ (compensatory head movements)	< 0.035 (4.3yrs) 1/60 (10yrs)	0.1 (4.3yrs) 1/20 (10yrs)	+1.5 (20mo) +2.6 (10yrs)	+2.5 (20mo) +1 (10yrs)	Moderate conc constr	Normal MRI (9mo)	-		

*RPE65* p.[**Arg296LysfsX7**]+[Splice defect]	LCA-50	F	+	1/10 (3yrs)	1/10 (3yrs)	+5 (8mo)	+5.75 (8mo)		Normal MRI (7mo)			

*GUCY2D* p. [Pro130LeufsX36 (+)Splice defect]	LCA-51	M	+	NLP	NLP							

*GUCY2D* p.[Phe565 Ser(+)Phe565Ser]	LCA-52	M	+	LP	LP	+4	+4		Normal MRI	-	US: hydro-uretero-nephrosis	

*GUCY2D* p.[Arg768Trp(+)Arg768Trp]	LCA-53	M										

*GUCY2D* p.[Arg768Trp]+[Phe565Ser]	LCA-54	F	+	1/600 (4.9yrs)	1/120 (4.9yrs)	+3.75 (4.7yrs)	+4.25 (4.7yrs)	Constricted (2.6yrs)		-		

*GUCY2D* p.[Arg76 8Trp]+[**Lys866Asn**]	LCA-55	M	+	LP	LP	+9	+9	10°		MR autism		

*GUCY2D* p.[**Glu196Val**]+[**Pro711Leu**]	LCA-56	F	+	1/30 (5.5yrs) 3/100 (23yrs)	1/30 (5.5yrs) 3/100 (23yrs)	+6	+6	Moderate conc constr		-		

*GUCY2D* p.Pro575 Leu	LCA-57	F	+	6/60	6/36	-6	-6		MRI: hypoplasia optic nerves	MR		

*AIPL1* p.[Trp278X]+[Trp278X]	LCA-58 (Yzer, Leroy et al. 2006)	F		<20/600 (3yrs) 1/50 (8 yrs)	<20/600 (3yrs) 1/50 (8 yrs)	+8	+8		Normal CT scan	-		

*AIPL1* p.[Trp278X (+)Trp278X]	LCA-59	M	+	<1/50 (2yrs) 1/100 (4.10yrs)	<1/50 (2yrs) 1/100 (4.10yrs)	+3.5D (2.5yrs)	?		Normal CT scan (6mo)	Mild Developmental delay		

*AIPL1* p.[Trp278X (+)Trp278X]	LCA-60	M										

*AIPL1* p.[Trp278X]+[Trp278X]	LCA-61	F	+	<1/100 (7yrs) LP with limited loc (21yrs)	<1/100 (7yrs) LP with limited loc (21yrs)	+6	+6	20° (7yrs) Residual pericentral remnants (21yrs)	Normal CT scan (10mo)	-		

*AIPL1* p.[Thr114 Ile; Pro376Ser]	LCA-62	F	+	1/10	1/10				Normal MRI	-		

*CRX* p.Tyr142 Cys	LCA-63	M	+	LP	LP	+9	+9			-		

*CRX* p.Val242 Met	LCA-64	M										

*RDH12* p.[Ala269 GlyfsX2] +[**Val233Asp**]	EORD-6	M	-	5/10 (4yrs) 1/10 (23yrs)	5/10 (4yrs) 1/10 (23yrs)			30° (8yrs) 5° (12yrs)	Normal CT scan	-		

*RDH12* p.[Ala269 GlyfsX2] +[**Ser175Leu**]	EORD-7	M	+	3/9 (3.5yrs) HM (19yrs)	3/9 (3.5yrs) 1.5/10 (19yrs)			OD: temporal crescent, OS: 70° (6yrs) OD: status-quo OS: central 5° (19.3yrs)		-		

*RDH12* p.[Ala269 GlyfsX2(+)Ala269 GlyfsX2]	EORD-8	M		4/10	4/10			10°				

*RPGRIP1* p.Arg890X	LCA-65	M		1/10	1/10			10°				

If available, the age of the first and last measurement is mentioned between brackets. A question mark indicates an uncertain status. Blank fields indicate features for which no information could be obtained. Clinical data on the two patients included in the Phase I clinical trial for *RPE65* gene-replacement therapy (LCA-47 and LCA-49) concern the period preceding therapy. Characteristics described in “Other features” are binocular, if not mentioned otherwise.

Abbreviations used: N°, number;; nyst, nystagmus; BCVA, best corrected visual acuity; OD, right eye; OS, left eye; ODS, both eyes; ERG, electroretinogram; VF, visual field; MTS, molar tooth sign; MRI, magnetic resonance imaging; MR, mental retardation; NPHP, nephronophtisis; SE, spherical equivalent; +, present; -, absent; yr(s), year(s); mo, month(s); W, week(s); REM, roving eye movements; HM, hand motion; LP, light perception; NLP, no light perception; CF, counting fingers; AF, autofluorescence; OCT, optical coherence tomography; US, ultrasound; SIDS, sudden infant death syndrome; conc constr, concentrically constricted; G, green; B, blue; R, red; O, orange; P, pink; RPE, retinal pigment epithelium; PPRPE, preserved para-arteriolar retinal pigment epithelium; astigm, astigmatism; loc, localization; CRD, cone-rod dystrophy; SCP, subcapsularis posterior; dev del, developmental delay; MEI, middle ear infections; RDI, renal diabetes insipidus; CKD5, renal failure; RTx, transplantation.
